# Divergent neurodegenerative patterns: Comparison of [^18^F] fluorodeoxyglucose-PET- and MRI-based Alzheimer’s disease subtypes

**DOI:** 10.1093/braincomms/fcae426

**Published:** 2024-11-23

**Authors:** Sophia H Wheatley, Rosaleena Mohanty, Konstantinos Poulakis, Fedor Levin, J Sebastian Muehlboeck, Agneta Nordberg, Michel J Grothe, Daniel Ferreira, Eric Westman

**Affiliations:** Division of Clinical Geriatrics, Department of Neurobiology, Care Sciences and Society, Center for Alzheimer Research, Karolinska Institutet, 171 77 Stockholm, Sweden; Division of Clinical Geriatrics, Department of Neurobiology, Care Sciences and Society, Center for Alzheimer Research, Karolinska Institutet, 171 77 Stockholm, Sweden; Division of Clinical Geriatrics, Department of Neurobiology, Care Sciences and Society, Center for Alzheimer Research, Karolinska Institutet, 171 77 Stockholm, Sweden; Deutsches Zentrum für Neurodegenerative Erkrankungen (DZNE), 18147 Rostock, Germany; Division of Clinical Geriatrics, Department of Neurobiology, Care Sciences and Society, Center for Alzheimer Research, Karolinska Institutet, 171 77 Stockholm, Sweden; Division of Clinical Geriatrics, Department of Neurobiology, Care Sciences and Society, Center for Alzheimer Research, Karolinska Institutet, 171 77 Stockholm, Sweden; Theme Inflammation and Aging, Karolinska University Hospital, 171 76 Stockholm, Sweden; Reina Sofia Alzheimer Centre, CIEN Foundation, ISCIII, 28031 Madrid, Spain; Division of Clinical Geriatrics, Department of Neurobiology, Care Sciences and Society, Center for Alzheimer Research, Karolinska Institutet, 171 77 Stockholm, Sweden; Facultad de Ciencias de la Salud, Universidad Fernando Pessoa Canarias, 35016 Las Palmas, España; Division of Clinical Geriatrics, Department of Neurobiology, Care Sciences and Society, Center for Alzheimer Research, Karolinska Institutet, 171 77 Stockholm, Sweden

**Keywords:** Alzheimer’s disease, data-driven models, subtypes, PET, MRI

## Abstract

[^18^F] fluorodeoxyglucose (FDG)-PET and MRI are key imaging markers for neurodegeneration in Alzheimer’s disease. It has been well established that parieto-temporal hypometabolism on FDG-PET is closely associated with medial temporal atrophy on MRI in Alzheimer’s disease. Substantial biological heterogeneity, expressed as distinct subtypes of hypometabolism or atrophy patterns, has been previously described in Alzheimer’s disease using data-driven and hypothesis-driven methods. However, the link between these two imaging modalities has not yet been explored in the context of Alzheimer’s disease subtypes. To investigate this link, the current study utilized FDG-PET and MRI scans from 180 amyloid-beta positive Alzheimer’s disease dementia patients, 339 amyloid-beta positive mild cognitive impairment and 176 amyloid-beta negative cognitively normal controls from the Alzheimer’s Disease Neuroimaging Initiative. Random forest hierarchical clustering, a data-driven model for identifying subtypes, was implemented in the two modalities: one with standard uptake value ratios and the other with grey matter volumes. Five hypometabolism- and atrophy-based subtypes were identified, exhibiting both cortical-predominant and limbic-predominant patterns although with differing percentages and clinical presentations. Three cortical-predominant hypometabolism subtypes found were Cortical Predominant (32%), Cortical Predominant+ (11%) and Cortical Predominant posterior (8%), and two limbic-predominant hypometabolism subtypes found were Limbic Predominant (36%) and Limbic Predominant frontal (13%). In addition, little atrophy (minimal) and widespread (diffuse) neurodegeneration subtypes were observed from the MRI data. The five atrophy subtypes found were Cortical Predominant (19%), Limbic Predominant (27%), Diffuse (29%), Diffuse+ (6%) and Minimal (19%). Inter-modality comparisons showed that all FDG-PET subtypes displayed medial temporal atrophy, whereas the distinct MRI subtypes showed topographically similar hypometabolic patterns. Further, allocations of FDG-PET and MRI subtypes were not consistent when compared at an individual level. Additional analysis comparing the data-driven clustering model with prior hypothesis-driven methods showed only partial agreement between these subtyping methods. FDG-PET subtypes had greater differences between limbic-predominant and cortical-predominant patterns, and MRI subtypes had greater differences in severity of atrophy. In conclusion, this study highlighted that Alzheimer’s disease subtypes identified using both FDG-PET and MRI capture distinct pathways showing cortical versus limbic predominance of neurodegeneration. However, the subtypes do not share a bidirectional relationship between modalities and are thus not interchangeable.

## Teaser

Wheatley *et al*. report distinct patterns of limbic versus cortical hypometabolism and atrophy in data-driven Alzheimer’s disease subtypes using [18F] fluorodeoxyglucose (FDG)-PET and MRI. However, these patterns were not consistent across both modalities, indicating that FDG-PET and MRI are not interchangeable at an individual level when assessing disease heterogeneity.

## Graphical Abstract

**Graphical Abstract:**
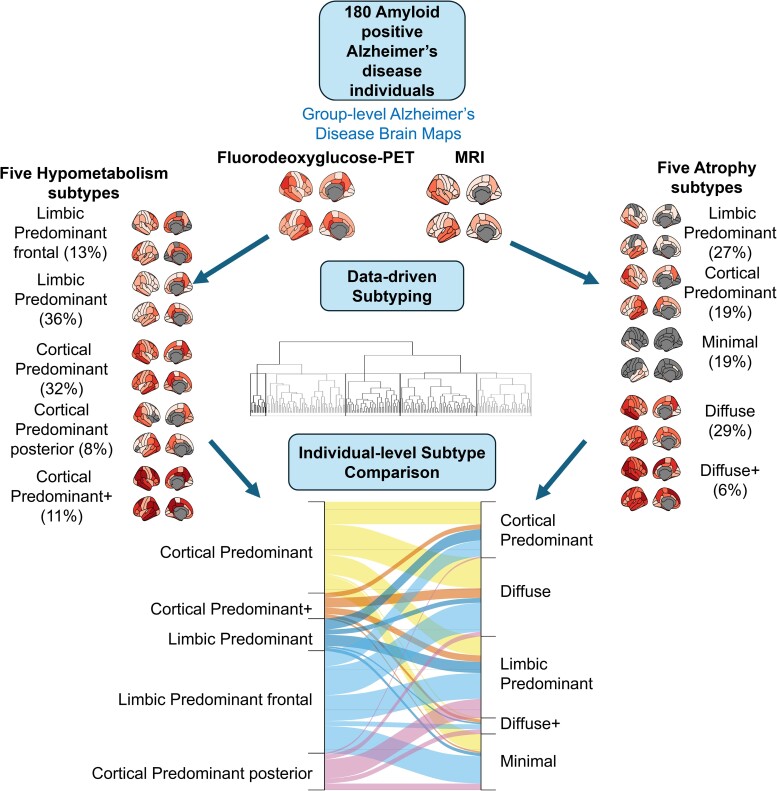

## Introduction

Alzheimer’s disease (Ad) is the most common neurodegenerative disease^1^ and is heterogeneous in nature.^2^ Over the last years, distinct Ad subtypes displaying different clinical and biological features have been identified.^2,3^ Despite this, there are discrepancies in findings across the studies, especially at an individual level.^4^ The common consensus from Ad subtyping research is the presence of distinct cortical-predominant versus limbic-predominant profiles of neurodegeneration, which primarily reflect differential distributions of tau neurofibrillary tangles pathology from neuropathological findings.

Corticolimbic subtypes were first identified from neuropathological data by distributions of neurofibrillary tangles in cortical and limbic regions. Such Ad subtypes were coined as ‘Hippocampal Sparing’ and ‘Limbic Predominant’ when compared with ‘Typical AD’.^5^ Typical Ad is described by a balanced distribution of neurofibrillary tangles in the cortical and limbic regions, whereas the other two subtypes are described at opposite ends of the spectrum, from low to high hippocampal neurofibrillary tangle load relative to neocortical regions. Similar hypothesis-driven approaches have been applied to *in vivo* methods such as structural MRI, where atrophy patterns resembled prior neuropathologically defined subtypes.^6,7^ Furthermore, Ad subtypes have been observed in data-driven studies using *in vivo* imaging.^2,8^ A framework was built based on tau pathology and atrophy in Ad subtype topography along two axes of ‘typicality’ and ‘severity’.^2^ ‘Typicality’ was proposed as a spectrum with ‘Limbic Predominant’ and ‘Hippocampal Sparing’ on opposite ends with ‘Typical AD’ in the middle, whereas ‘severity’ reflects findings from atrophy-based studies with ‘Minimal’ (low atrophy) and ‘Diffuse’ (high atrophy) patterns as extremes compared with ‘Typical Ad.’ It is not certain yet whether this framework could be applied to imaging modalities other than tau-PET and MRI-based atrophy. Furthermore, longitudinal MRI studies of Ad subtypes revealed that neurodegeneration progressed along either a cortical or a limbic pathway.^9^ Similarly, modelling the spread of tau pathology in Ad, two pathways were proposed starting either in the entorhinal cortex or in association cortices.^10^

Molecular imaging complements MRI by measuring functional changes for the study of neuropathological hallmarks of AD. However, the main body of research in AD heterogeneity has used MRI scans, although research is branching out to other imaging modalities. There are few studies investigating AD heterogeneity using [^18^F] fluorodeoxyglucose (FDG)-PET. Only one data-driven study has been published using a data-driven approach to FDG-PET to find different patterns of hypometabolism in AD,^11^ which has been complemented by a recent study in amnestic mild cognitive impairment (MCI) individuals.^12^ Similar spatial subtypes to those found in MRI studies were identified, including a ‘Cortical Predominant’ hypometabolism subtype showing similarity to the Hippocampal Sparing atrophy subtype, and a ‘Limbic Predominant’ subtype. An important aspect to consider for linking the findings in these FDG-PET studies with MRI studies is the use of the term ‘Typical AD’ pattern, which differs across the two modalities. Prominent parieto-temporal hypometabolism reflects the typical AD pattern in FDG-PET, while widespread atrophy in the cortex and hippocampus reflects the typical AD pattern in MRI.^13-15^

Comparisons of FDG-PET and MRI across the AD continuum have been performed.^16-19^ These comparative studies found correlations across the two modalities, but not a full correspondence of regional neurodegeneration. Similarly, comparison of hypometabolism patterns in amnestic MCI individuals was assessed relative to hippocampal atrophy.^12^ There have also been studies investigating the relationship between MRI and tau-PET that show that the neurodegeneration patterns do not always correspond between the modalities.^4,20-24^ Exploration of the mismatch between tau uptake and hypometabolism in AD, MCI and cognitively normal (CN) individuals has also highlighted the complexity of the relationship between modalities presumed to be associated.^25^ However, the combination of FDG-PET and MRI techniques encompassing regions beyond the hippocampus in AD subtypes has not yet been performed.

The aim of the current study was to simultaneously evaluate heterogeneity in measures of neurodegeneration (FDG-PET and MRI) to: (i) identify data-driven subtypes of AD, thereby testing the existence of corticolimbic pathways, (ii) investigate the overlap and relationship of these corticolimbic subtypes across FDG-PET and MRI and (iii) compare data-driven with hypothesis-driven methods of subtyping. Corresponding neurodegeneration patterns and clinical and demographic information were assessed for each of the subtypes. Understanding the link between atrophy and hypometabolism could lead to better subtype classification in the context of a biological framework of AD.

## Materials and methods

### Participants

The cohort consisted of 180 amyloid-beta positive (Aβ+) AD dementia, 339 Aβ+ MCI and 176 amyloid-beta negative (Aβ−) CN individuals with both a MRI and a FDG-PET scan, from the Alzheimer’s Disease Neuroimaging Initiative (ADNI), including ADNI1, ADNI2/GO and ADNI3 phases (Table 1). The ADNI is a longitudinal multi-centre study aimed at investigating whether neuroimaging methods, together with genetic, clinical and neuropsychological measures, could be used to follow the progression of MCI and AD. The ADNI was launched in 2003 as a public–private partnership, led by principal investigator Michael W. Weiner, MD. For AD patients, the inclusion criteria consisted of: Mini-Mental State Examination scores of 20–26, Clinical Dementia Rating (CDR) scores of 0.5 or 1.0 and meeting the criteria for probable AD. More information of the inclusion and exclusion criteria for the participants can be found on the ADNI website (https://adni.loni.usc.edu/help-faqs/adni-documentation/). FDG-PET scans from the initial visit and corresponding MRI scans had a mean of 25-day interval and a maximum of 156-day interval (Supplementary Fig. 1). Individuals with AD and MCI were included in this study based on Aβ positivity and CNs on Aβ negativity. This was first determined by an AV45-PET standard uptake value ratio (SUVR) >1.11.^26^ If these data were not available for the individual, we used their Aβ (1–42) values from CSF, which were deemed Aβ positive if <880pg/ml.^27^

**Table 1:** Cohort demographics and clinical characteristics

Clinical and demographic characteristics	AD dementia	MCI	CN	*P*-values
*N*	180	339	176	—
Women (%)	81 (45%)	145 (43%)	84 (48%)	0.558
Age (years)	74 (8)	73 (7)	75 (7)	0.245
Disease duration (years)	3 (3)	—	—	—
Education (years)	15 (3)	16 (3)	17 (3)	<0.001
*APOE* ɛ4 (%)	126 (70%)	200 (59%)	34 (19%)	<0.001
MMSE	23 (2)	27 (3)	29 (1)	<0.001
Global CDR	0.8 (3)	0.6 (0.2)	0.03 (0.2)	<0.001
ADNI-EF	−0.4 (0.7)	0.3 (0.6)	0.8 (0.5)	<0.001
ADNI-MEM	−0.8 (0.3)	0.03 (0.6)	0.9 (0.5)	<0.001
ADNI-LAN	−0.2 (0.6)	0.3 (0.5)	0.8 (0.5)	<0.001
CDF t-tau (pg/ml)	383 (149)	335 (138)	223 (70)	<0.001
CSF p-tau (pg/ml)	38 (16)	33 (15)	20 (6)	<0.001
CSF Aβ (pg/ml)	587 (191)	723 (234)	1204 (333)	<0.001

The values shown in the table are the means with standard deviations in brackets except for number of individuals, women and *APOE* ɛ4 carriership for which the percentages are provided. The *P*-values correspond to the χ^2^ tests conducted for categorical variables and Kruskal–Wallis for continuous variables between AD, MCI and CN groups. Values were corrected for multiple comparison with the Holm–Šidák method.

AD, Alzheimer’s disease individuals; CN, cognitively normal individuals; *APOE* ɛ4, apolipoprotein E ɛ4 allele; MMSE, Mini-Mental State Examination; CDR, The Clinical Dementia Rating Scale; ADNI-EF, Alzheimer’s Disease Neuroimaging Initiative executive function composite score; ADNI-MEM, Alzheimer’s Disease Neuroimaging Initiative memory composite score; ADNI-LAN, Alzheimer’s Disease Neuroimaging Initiative language composite score; CSF t-tau, total tau; CSF p-tau, phosphorylated tau; CSF Aβ, amyloid-beta 1–42 peptide.

### Fluorodeoxyglucose-PET

Multiple scanners were used for the FDG-PET scans and followed the appropriate protocols (https://adni.loni.usc.edu/help-faqs/adni-documentation/). Dynamic 3D scans made up of six 5-min frames were retrieved 30–60 min after administration of [^18^F] FDG. FDG-PET scans were co-registered to individuals’ MRI scans in Montreal Neurological Institute space using PETSurfer,^28,29^ and regional SUVRs were extracted without partial volume correction.^19,30^ We investigated 82 bilateral regions of interest (ROIs) defined on individual’s MRI including cortical and subcortical structures based on the standard atlases provided by FreeSurfer.^31,32^ From these regions, SUVRs were extracted in Montreal Neurological Institute space. Intensity normalization was carried out by dividing the regional values by the individual’s global mean uptake.^33^ This type of normalization has been used in prior FDG-PET dementia and subtyping studies.^11,34^

### MRI

T_1_-weighted magnetization-prepared rapid gradient echo MRI scans were preprocessed using FreeSurfer (version 6.0.0, https://freesurfer.net). MRI scans were collected from various sites using multiple scanners (both 1.5 and 3 T scanners), following the appropriate protocol (https://adni.loni.usc.edu/help-faqs/adni-documentation/). These data were preprocessed in-house through theHiveDB database.^35^ Briefly, the preprocessing pipeline involved removal of artefacts, transformation to Talairach space and segmentation of cortical and subcortical regions. From the same 82 regions used for the FDG-PET, cortical and subcortical grey matter volumes were extracted. In addition, we extracted white matter hyperintensity (WMH) volume measures from ADNI as a measure of small vessel disease. WMH volumes were extracted using proton density, T_1_ and T_2_ MRIs for ADNI1^36^ and segmentation of high-resolution 3D T_1_ and FLAIR sequences with a Bayesian approach for ADNI-GO/2.^37^

The raw MRI scans were assessed visually for quality control. Additionally, the estimated total intracranial volumes (ICV) from the FreeSurfer output were plotted against the regional volumes to identify any outliers where the values were grossly under- or over-estimated. WMH volumes were adjusted for ICV values using the same method, but with the ICV values from the WMH datasheet extracted from ADNI. The raw images were then checked for these cases to confirm exclusion. Further, scans that were not segmented or registered properly were excluded. In total, 29 individuals (6 CN and 23 AD) were excluded due to poor PET scan quality, failed quality control, poor segmentation or under-/over-estimated ICV.

The grey matter volumes were adjusted for head size per region by using a residual approach using the ICV values from the CN individuals^38,39^ as shown below:


$$\mathrm{Volum}e_{\mathrm{adj}}=\mathrm{Volum}e_{\mathrm{raw}}\text{–}\beta (\mathrm{IC}V_{\mathrm{raw}}\text{–}\mathrm{IC}V_{\mathrm{mean}})$$

This adjustment was carried out for each group (CN and AD) in relation to the CNs. Volume_raw_ is the uncorrected volume for the brain ROI. The β value and mean ICV, ICV_mean_, are calculated by running a linear regression model per ROI in relation to the ICV using data from the CNs.

### CSF biomarkers

In addition to CSF Aβ (1–42) values, we assessed CSF measures of tau phosphorylated at threonine 181 (p-tau) and total tau (t-tau) taken from an automated Elecsys cobas e 601 analyser.

### Neuropsychological testing

We used the Mini-Mental State Examination (MMSE) to assess global cognition and composite scores for executive function, memory and language^40-42^ to assess specific cognitive domains. For MMSE, lower scores indicate higher global cognitive impairment and lower scores for each of the composite scores indicate greater impairment.

### Data-driven subtyping: hierarchical clustering analysis

To classify individuals into subtypes in FDG-PET and MRI, unsupervised random forest hierarchical clustering was performed (Supplementary Figs 2 and 3) to identify the linear and non-linear relationships from the regional values. This method has previously been used to investigate heterogeneity within neurodegenerative diseases using grey matter volumes.^10,43,44^ In the current study, an identical clustering procedure was applied to regional glucose metabolism values and grey matter volumes using the same 82 bilateral cortical and subcortical ROIs. Two separate clustering models were performed, one using glucose metabolism values and another with grey matter volumes. In addition, to the AD dementia sample, we performed a validation of the data-driven clustering using the MCI sample as an external data set.

First, a distance matrix was calculated based on the regional values using a random forest algorithm, which provides information on the similarities and dissimilarities across the brain regions. The optimization of hyperparameters was performed by selecting the values with the lowest out-of-bag errors^45^ for: number of variables randomly sampled at each split (*mtry*) and minimum size of the terminal nodes (*nodesize*). The number of trees was set to 20 000 for all models. Additionally, the stability of the chosen random forest model was tested by running the random forest algorithm 100 times. The differences between the chosen model and the simulated models were calculated (Supplementary Figs 4 and 5).

The distance matrix was then reduced to three dimensions using classical multi-dimensional scaling to simplify the interpretation of the most important features that distinguish the clusters from each other. The first three dimensions from the multi-dimensional scaling were used for clustering as they explained the greatest differences between the groups, identified by plotting the eigenvalues of the dimensions. Agglomerative hierarchical clustering with average linkage was then run using the reduced matrix to identify clusters. The output of the clustering is a dendrogram, and to group the individuals into subtypes, the number of clusters needs to be chosen. This number (*k*) was derived by using various cluster validation indices, namely the Calinski–Harabasz, Davies–Bouldin, Dunn and Silhouette indices from *NbClust* and *fpc* libraries in R. The Calinski–Harabasz index is calculated by comparing the between-cluster variance with the within-cluster variance. The Davies–Bouldin index evaluates cluster compactness and distinctness by comparing within-cluster distances to between-cluster distances. Similarly, the Dunn index assesses cluster compactness and separation. The Silhouette index is the measure of how well the objects fit into its allocated cluster. Collectively, these four indices capture a well-rounded assessment for choosing the number of clusters for our models.

### Inter-modality comparison of subtypes

Comparisons between FDG-PET and MRI subtypes were investigated by: (i) subtyping in one modality and mapping the corresponding atrophy/hypometabolism patterns in the other modality, (ii) frequency of the derived FDG-PET and MRI subtypes and (iii) crossover between individual subtype allocations.

Regional atrophy patterns of the FDG-PET subtypes and regional hypometabolism patterns of the MRI subtypes were assessed using *w*-scores. *W*-scores^17^ were calculated as *z*-scores by subtracting the mean and dividing by the standard deviation from the CN data and adjusted for covariates: age, sex, education and *APOE* ɛ4 allele carriership. Furthermore, the frequencies of the individuals belonging to a given subtype in both modalities, e.g. an individual being classified as Cortical Predominant in FDG-PET clustering and Cortical Predominant in MRI clustering, were calculated. The different modality-specific subtypes were compared in terms of the defining topographical patterns, which enabled us to visualize the similarities and differences across the subtypes and whether individuals had similar neurodegeneration patterns in the two modalities. To test whether the modality-specific subtype classifications overlapped at an individual level, the individuals’ subtype allocations were compared in an alluvial plot. Additionally, to numerically demonstrate the overlap, a ratio for each FDG-PET and MRI subtype pairing was calculated using the total number of cases for each FDG-PET subtype. For instance, the ratio was determined by dividing the number of Cortical Predominant MRI cases by the total number of Cortical Predominant FDG-PET cases.

### Data-driven versus hypothesis-driven subtypes

This analysis was performed to compare our data-driven method with a previously described hypothesis-driven method designed to identify neuropathologically defined corticolimbic subtypes.^5-7^ The aim was to investigate whether there would be an overlap in the subtype categorization from these two methods. Given that hypothesis-driven subtyping has been applied to MRI data previously, this analysis additionally aimed to determine whether such a subtyping could be adapted using FDG-PET data, mirroring techniques previously applied to tau-PET.^46^ Prior hypothesis-driven studies have used the hippocampus-to-cortex ratio to group AD individuals into one of three subtypes (‘Limbic Predominant’, ‘Typical AD’ and ‘Hippocampal Sparing’) using a two-step process using the 75th and 25th percentiles and median hippocampal and cortical values.^5-7^ Hypothesis-driven subtype labels in the current study were ‘Limbic Predominant’ (low hippocampus-to-cortex ratio) and ‘Cortical Predominant’ (high hippocampus-to-cortex ratio).

The hippocampus-to-cortex ratio captures whether a subtype shows either a limbic or cortical pattern and was conceptualized as the ‘typicality’ axis in a recent framework explaining the topography of AD subtypes.^2^ A second axis of the subtypes was termed ‘severity’, which refers whether a subtype shows atypically low or high atrophy. Hence, in this analysis, we further compared the data-driven and hypothesis-driven subtyping in the context of the published framework. For MRI, the measure for ‘typicality’ was the hippocampus-to-cortex ratio from volume measures and the measure for ‘severity’ was the total grey matter volume. For FDG-PET, ‘typicality’ was hippocampus-to-cortex ratio from glucose uptake and ‘severity’ was the total average cortical SUVR. All subtype classifications were then plotted along these two axes. These are orthogonal to each other, explaining the differing patterns in the five common patterns of atrophy (Hippocampal Sparing, Limbic Predominant, Typical AD, Minimal and Diffuse). To explore this, Pearson’s correlations were calculated for measures of ‘typicality’ and ‘severity’ within and between the two imaging modalities. Typicality and severity measures were further used to generate a conceptual figure for the two modalities’ subtypes along these two axes. Values for each subtype were rescaled to range from values 0 to 1 using min–max normalization. These normalized values where then averaged and plotted.

### Statistical analysis

RStudio and R version 4.2.0 were used for statistical analyses. *W*-scores were plotted to show the differences in the regional SUVRs and grey matter volumes in the subtypes compared with the CN individuals.^17,47^ For FDG-PET, the *w*-scores were calculated using SUVRs scaled by the pons as the reference region. The pons was chosen because it has been compared with other reference regions across ageing and AD studies using FDG-PET and it has been shown to work well with both partial volume corrected and non-partial volume corrected data.^48^ These values were averaged across the AD individuals, respectively, and reversed so that the higher *w*-scores correspond to greater neurodegeneration in the AD group. Brain maps were created using the *ggseg* library in R.^49^

The demographic and clinical variables of the subtypes were compared using χ² and Kruskal–Wallis tests that were adjusted for multiple comparisons with the Holm–Šidák method (missing values are listed in Supplementary Table 1). Significance was deemed if the *P*-values were <0.05 where the null hypothesis can be rejected when P<α with α = 0.001, this stringent alpha value was set to avoid Type 1 family-wise errors. Comparisons were made between the subtypes and the CN individuals, as well as pairwise comparisons between the groups.

## Results

### Cohort characteristics

The basic clinical and demographic information of the cohort is reported in Table 1. As expected, The AD, MCI and CN groups significantly differed in terms of percentage of *APOE* ɛ4 carriers, cognitive measures and CSF markers. There were no significant differences between groups in terms of sex and age.

### FDG-PET clustering

**Figure 1:**
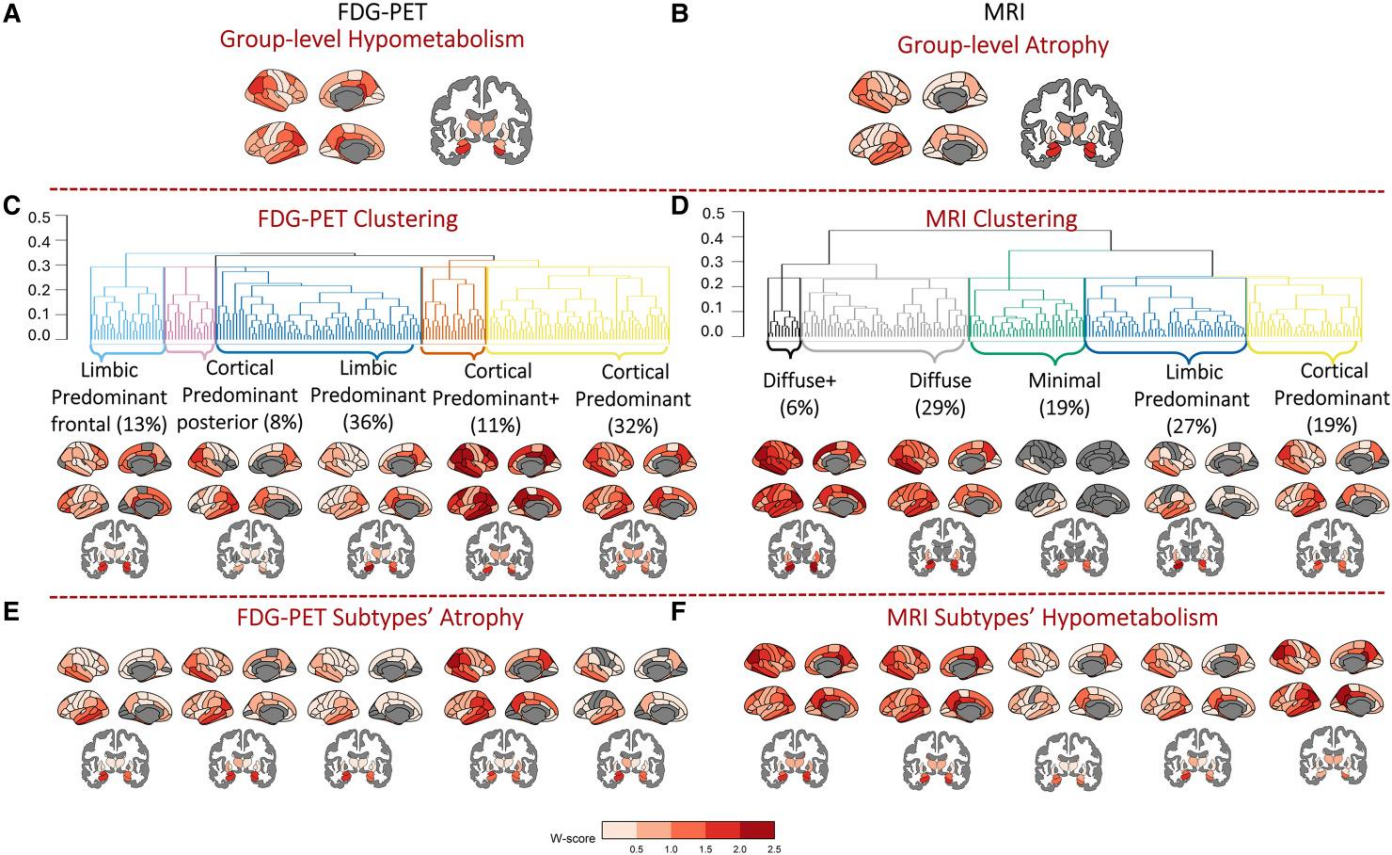
**Data-driven AD subtypes based on FDG-PET and MRI.** Overall patterns of neurodegeneration in Aβ+ AD versus Aβ− CNs visualized for (**A**) hypometabolism in FDG-PET and (**B**) atrophy in MRI. Random forest hierarchical clustering identified five subtypes of each (**C**) hypometabolism and (**D**) atrophy, which are shown by dendrograms (Euclidean distance between clusters on *y*-axis) and brain maps in each modality. Corresponding patterns of (**E**) atrophy patterns in FDG-PET subtypes and (**F**) hypometabolism patterns in MRI subtypes were visualized. All brain maps (**A–F**) are represented as *w*-scores where regional values (pons scaled SUVR in FDG-PET and volumes in MRI) are adjusted for age, sex, education and *APOE* ɛ4 carriership.

The overall AD group’s hypometabolism pattern showed neurodegeneration in posterior cingulate and frontal cortical regions and deeper structures such as the hippocampus (Fig. 1A). The clustering model resulted in two main distinguishing patterns, neurodegeneration in cortical versus limbic pathways (Fig. 1C). The Calinski–Harabasz index peaked at five clusters, for the Davies–Bouldin index was lower at five, the Dunn index plateaued at five and the Silhouette index was highest at 3–5 clusters (Supplementary Fig. 6). Therefore, we chose five clusters as the optimal solution for the FDG-PET model. The FDG-PET model was split into five distinct hypometabolism-based subtypes. Three subtypes showed cortical-predominant hypometabolism of differing severity and spatial distribution. The Cortical Predominant posterior subtype had cortical hypometabolism mainly in the posterior regions (8%), whereas the Cortical Predominant and Cortical Predominant+ subtypes showed more widespread cortical hypometabolism (32 and 11%, respectively). The Cortical Predominant+ subtype had greater hypometabolism than the other two cortical-predominant subtypes. Although all the subtypes showed some hypometabolism in the hippocampus, these subtypes had proportionally less involvement of this region compared with the cortical areas. Two subtypes displayed limbic hypometabolism, focal to the medial temporal and deeper structures (amygdala, hippocampus). Here, a principal Limbic Predominant subtype (36%) could be distinguished from a Limbic Predominant frontal subtype (13%). In the clustering dendrogram (Fig. 1C), the Limbic Predominant frontal cluster originates from its own branch, whereas the Limbic Predominant cluster comes from the same branch as the Cortical Predominant posterior cluster. Thus, the clustering separates these subtypes by a frontal versus a posterior hypometabolism pattern. By contrast, the Cortical Predominant and Cortical Predominant+ clusters are separated on the opposite side of the dendrogram by the severity of their cortical hypometabolic patterns. Our FDG-PET MCI model also showed a split between one limbic-predominant and two cortical-predominant subtype patterns (Supplementary Fig. 10C). Again, FDG-PET patterns are more widespread compared with the MRI patterns even at the prodromal stage (Supplementary Fig. 10C-D).

For inter-modality comparisons, the corresponding atrophy patterns in these subtypes were plotted (Fig. 1E). Based on visual comparison of the *w*-scores, the brain maps were topographically similar across FDG-PET subtypes, showing typical AD atrophy in medial temporal, hippocampal and some frontal areas. However, the atrophy pattern of the Cortical Predominant subtype was not as widespread in the cortical regions compared with the hypometabolism. Additional maps using partial volume corrected SUVRs from PETSurfer were plotted (Supplementary Fig. 8). These brain maps did not differ greatly from our maps in Fig. 1 topographically but did result in lower *w*-scores.

Regarding demographic and clinical differences among the FDG-PET AD subtypes (Table 2), the Cortical Predominant+ subtype was the youngest (67 years) and had the earliest age at onset (65 years), more pronounced language impairment and lowest executive function scores. This subtype also had the highest grey matter volume-based and SUVR-based hippocampus-to-cortex ratios. The other two cortical subtypes (Cortical Predominant, Cortical Predominant posterior) had a higher SUVR-based hippocampus-to-cortex ratio than the limbic subtypes. Cortical Predominant posterior also had a high hippocampus-to-cortex ratio using grey matter volumes compared with the limbic subtypes. Among the limbic subtypes, Limbic Predominant frontal was the oldest and had latest age at onset and worst language scores. There were no significant differences between the subtypes for the other variables: sex, disease duration, years of education, *APOE* ɛ4 carriers, MMSE, CDR, cognitive measures of memory, CSF biomarkers and total WMH volumes. Although not statistically significant, two of the cortical subtypes, Cortical Predominant and Cortical Predominant+, had lower percentage of *APOE* ɛ4 carriers and Limbic Predominant frontal the highest percentage of *APOE* ɛ4 carriers.

**Table 2:** Demographic and clinical characteristics of the FDG-PET AD subtypes

Demographic and clinical characteristics	Cortical Predominant	Cortical Predominant+	Limbic Predominant	Limbic Predominant frontal	Cortical Predominant posterior	Cognitively Normal	*P*-values
*N* (%)	57 (32%)	20 (11%)	64 (36%)	23 (13%)	16 (8%)	176	—
Women (%)	22 (39%)	10 (50%)	32 (50%)	10 (43%)	7 (44%)	84 (48%)	0.768
Age (years)	75 (7.9)	67 (8.8)	74 (6.8)	78 (6.6)	73 (8.8)	75 (7.1)	<0.001^a-d^
Disease duration (years)	2.5 (2.7)	2.7 (2.1)	2.9 (2.5)	3.1 (2.9)	1.8 (2.1)	—	0.274
Age at onset (years)	72 (8.1)	65 (8.3)	71 (7)	75 (7.4)	71 (8.8)	—	<0.001^a-c^
Education (years)	15 (2.5)	16 (2.8)	16 (2.9)	15 (3.5)	15 (2.2)	17 (2.6)	0.361
*APOE* ɛ4 (%)	35 (61%)	11 (55%)	48 (75%)	20 (87%)	12 (75%)	34 (19%)	0.083
MMSE	23 (2.1)	22 (2.6)	24 (2)	23 (1.9)	23 (1.7)	29 (1.4)	0.167
Global CDR	0.77 (0.25)	0.88 (0.22)	0.73 (0.25)	0.77 (0.25)	0.89 (0.4)	0.025 (0.16)	0.225
ADNI-EF	−0.43 (0.62)	−0.99 (0.73)	−0.19 (0.61)	−0.6 (0.51)	−0.58 (0.7)	0.8 (0.47)	<0.001^a,b,e^
ADNI-MEM	−0.85 (0.32)	−0.89 (0.32)	−0.66 (0.34)	−0.93 (0.31)	−0.8 (0.4)	0.89 (0.52)	0.007
ADNI-LAN	−0.24 (0.53)	−0.43 (0.52)	0.041 (0.54)	−0.5 (0.61)	−0.31 (0.56)	0.82 (0.52)	<0.001^a,e,f^
CSF t-tau (pg/ml)	378 (145)	407 (143)	415 (167)	335 (98)	335 (152)	223 (70)	0.958
CSF p-tau (pg/ml)	38 (15)	41 (16)	43 (19)	33 (10)	33 (15)	20 (6.3)	0.961
CSF Aβ (pg/ml)	585 (248)	588 (173)	588 (169)	596 (150)	578 (139)	1204 (333)	0.537
SUVR-based hippocampus-to-cortex ratio	0.35 (0.04)	0.38 (0.047)	0.3 (0.034)	0.32 (0.032)	0.33 (0.032)	0.32 (0.029)	<0.001^e,g,i,j,k,l^
Grey matter volume-based hippocampus-to-cortex ratio	0.16 (0.021)	0.19 (0.029)	0.16 (0.02)	0.16 (0.023)	0.17 (0.025)	0.18 (0.021)	<0.001^g,h,m^
Total grey matter volume (mm³)	558 154 (33 242)	544 820 (35 401)	554 040 (38 026)	537 830 (47 861)	539 736 (36 515)	580 718 (40 407)	0.129
Total average cortical uptake (SUVR)	1.5 (0.16)	1.5 (0.12)	1.6 (0.18)	1.6 (0.11)	1.6 (0.2)	1.7 (0.19)	0.021
Mean min–max normalized hippocampus-to-cortex ratio	0.64	0.82	0.29	0.43	0.53	—	—
Mean min–max normalized total average cortical uptake	0.67	0.67	0.58	0.64	0.58	—	—
Total WMH volume (ICV adjusted)	9.6 (9.4)	7.7 (13.1)	8 (7.7)	5.9 (6)	3.7 (2.4)	3.67 (4)	0.030

The values shown in the table are the means with standard deviations in brackets except for number of individuals, women and *APOE* ɛ4 for which the percentages are provided. The reported *P*-values correspond to χ^2^ tests which were used for categorical variables and Kruskal–Wallis for continuous variables and were corrected for multiple comparison with the Holm–Šidák method. Footnotes indicate cases where *P*-values were significant in the *post hoc* pairwise comparisons across AD subtypes, *P* < 0.05. The CN group data are displayed for reference.

*APOE* ɛ4, apolipoprotein E ɛ4 allele; MMSE, Mini-Mental State Examination; CDR, The Clinical Dementia Rating Scale; ADNI-EF, Alzheimer’s Disease Neuroimaging Initiative executive function composite score; ADNI-MEM, Alzheimer’s Disease Neuroimaging Initiative memory composite score; ADNI-LAN, Alzheimer’s Disease Neuroimaging Initiative language composite score; CSF t-tau, total tau; CSF p-tau, phosphorylated tau; CSF Aβ, amyloid-beta 1–42 peptide; WMH, white matter hyperintensity; ICV, estimated total intracranial volume.

^a^Cortical Predominant+ < Limbic Predominant, *P* < 0.05.

^b^Cortical Predominant+ < Cortical Predominant, *P* < 0.05.

^c^Cortical Predominant+ < Limbic Predominant frontal, *P* < 0.05.

^d^Limbic Predominant < Limbic Predominant frontal, *P* < 0.05.

^e^Limbic Predominant frontal < Limbic Predominant, *P* < 0.05.

^f^Cortical Predominant < Limbic Predominant, *P* < 0.05.

^g^Limbic Predominant < Cortical Predominant+, *P* < 0.05.

^h^Limbic Predominant frontal < Cortical Predominant+, *P* < 0.05.

^i^Limbic Predominant < Cortical Predominant, *P* < 0.05.

^j^Limbic Predominant < Cortical Predominant posterior, *P* < 0.05.

^k^Limbic Predominant frontal < Cortical Predominant, *P* < 0.05.

^l^Cortical Predominant posterior < Cortical Predominant+, *P* < 0.05.

^m^Cortical Predominant < Cortical Predominant+, *P* < 0.05.

### MRI clustering

The overall AD group showed atrophy in the expected medial temporal regions such as hippocampus and amygdala (Fig. 1B), which will be referred to as a ‘typical’ AD pattern for MRI. Similar to FDG-PET subtypes, clustering revealed a distinction between either a limbic or a cortical pathway in MRI (Fig. 1D). The Calinski–Harabasz index peaked at three, but was still high at five clusters, the Davies–Bouldin index was lower at five, the Dunn index was high for five albeit plateaued at six before a sharp increase after that and the Silhouette index was highest at three to six clusters (Supplementary Fig. 7). Therefore, we chose five clusters as the optimal solution for the MRI model based on these results and considering prior work identifying five biological subtypes. Another reason for choosing a higher cluster solution was based on the lack of sensitivity for finding atypical patterns when implementing a three- and four-cluster solutions. The MRI clustering model was split into five atrophy-based subtypes. In contrast to the FDG-PET subtypes which were limited to cortical and limbic subtypes, the MRI subtypes showed additional ‘minimal’ versus ‘diffuse’ atrophy patterns. Similar to the FDG-PET Cortical Predominant subtypes, a Cortical Predominant MRI subtype (19%) showed greater cortical atrophy relative to the hippocampus. The Limbic Predominant subtype (27%) had the opposite pattern with greater atrophy in the hippocampus relative to the cortex. The Minimal subtype (19%) had some atrophy in the hippocampus and amygdala, but very little atrophy compared with CN individuals in the cortical regions. There were two diffuse atrophy subtypes, one with greater overall atrophy, Diffuse+ (6%), and one with similarly diffuse but less severe atrophy (29%). Our MRI MCI model showed a split between a minimal atrophy and two limbic-predominant subtypes, with less distinct differences in severity (Supplementary Fig. 10D). Overall, the MCI data shows similarities with our AD-based models, but with weaker patterns due to early stage of the disease.

Based on the inter-modality comparison, the atrophy-based subtypes displayed hypometabolism of differing severity in temporo-parietal and lateral temporal regions often described to be the ‘typical AD’ pattern in FDG-PET scans (Fig. 1F). Compared with the corresponding atrophy maps of the FDG-PET subtypes (Fig. 1E), the hypometabolism maps (Fig. 1F, Supplementary Fig. 9) were more topographically similar to the MRI subtypes when based on visual comparison of *w*-scores. These corresponding maps showed both more pronounced (higher *w*-scores) and more widespread hypometabolism in the Minimal and Cortical Predominant subtypes compared with their atrophy maps (Fig. 1F, Supplementary Fig. 9).

Regarding demographic and clinical differences, among the MRI AD subtypes (Table 3), the Diffuse subtype had the lowest executive function scores compared with the Minimal and Limbic Predominant subtypes. Diffuse, Diffuse+ and Cortical Predominant had significantly worse executive function scores compared with the Minimal subtype. Minimal and Limbic Predominant subtypes had significantly lower SUVR-based hippocampus-to-cortex ratios to Cortical Predominant. Significant differences were also found in the grey matter volume-based hippocampus-to-cortex ratios: Cortical Predominant had the highest hippocampus-to-cortex ratio. There were no significant differences between the subtypes for the other variables: sex, age, disease duration, age at onset, years of education, *APOE* ɛ4 carriers, MMSE, CDR, cognitive measure of memory and language, CSF biomarkers and total WMH volumes. Despite not showing a significant difference, Diffuse+ had the highest proportion of *APOE* ɛ4 carriers (90%) and was the oldest group (79 years).

**Table 3:** Demographic and clinical characteristics of the MRI AD subtypes

Demographic and clinical characteristics	Cortical Predominant	Diffuse	Limbic Predominant	Diffuse+	Minimal	Cognitively Normal	*P*-values
*N* (%)	35 (19%)	51 (29%)	49 (27%)	10 (6%)	35 (19%)	176	—
Women (%)	10 (29%)	30 (59%)	22 (45%)	6 (60%)	13 (37%)	84 (48%)	0.049
Age (years)	70 (9.5)	74 (8.4)	76 (6.3)	79 (4.9)	73 (6.7)	75 (7.1)	0.004
Disease duration (years)	2.2 (2.3)	3.4 (2.5)	2.5 (2.7)	4.4 (3.2)	1.9 (2.1)	—	0.002
Age at onset (years)	68 (9.4)	71 (8.6)	74 (6.7)	75 (7.3)	71 (6.7)	—	0.021
Education (years)	15 (3)	15 (2.9)	16 (2.6)	16 (2.7)	16 (2.6)	17 (2.6)	0.197
*APOE* ɛ4 (%)	22 (63%)	38 (75%)	31 (63%)	9 (90%)	26 (74%)	34 (19%)	0.329
MMSE	23 (2.4)	23 (2)	23 (2.1)	23 (2.1)	24 (2.1)	29 (1.4)	0.767
Global CDR	0.79 (0.25)	0.83 (0.24)	0.74 (0.32)	0.89 (0.22)	0.72 (0.25)	0.025 (0.16)	0.121
ADNI-EF	−0.6 (0.62)	−0.72 (0.6)	−0.32 (0.5)	−0.63 (0.72)	−0.0096 (0.74)	0.8 (0.47)	<0.001^a-d^
ADNI-MEM	−0.78 (0.32)	−0.91 (0.33)	−0.78 (0.29)	−0.85 (0.43)	−0.65 (0.4)	0.89 (0.52)	0.059
ADNI-LAN	−0.22 (0.41)	−0.4 (0.52)	−0.22 (0.55)	−0.13 (0.67)	0.11 (0.68)	0.82 (0.52)	0.007
CSF t-tau (pg/ml)	390 (164)	398 (145)	380 (141)	280 (77)	377 (161)	223 (70)	0.065
CSF p-tau (pg/ml)	39 (17)	40 (16)	37 (14)	26 (7.4)	39 (18)	20 (6.3)	0.041
CSF Aβ (pg/ml)	564 (168)	575 (159)	661 (245)	536 (128)	551 (181)	1204 (333)	0.425
SUVR-based hippocampus-to-cortex ratio	0.35 (0.047)	0.34 (0.048)	0.32 (0.038)	0.34 (0.053)	0.31 (0.043)	0.32 (0.029)	<0.001^e,f^
Grey matter volume-based hippocampus-to-cortex ratio	0.18 (0.021)	0.17 (0.026)	0.15 (0.018)	0.16 (0.027)	0.15 (0.022)	0.18 (0.021)	<0.001^e,f,h,i^
Total grey matter volume (mm³)	561 400 (17 261)	518 539 (12 671)	552 539 (15 299)	478 411 (11 842)	606 360 (16 474)	580 718 (40 407)	<0.001^a-d,g,j-l^
Total average cortical uptake (SUVR)	1.6 (0.16)	1.5 (0.14)	1.6 (0.15)	1.4 (0.081)	1.7 (0.21)	1.7 (0.19)	<0.001^a,d^
Mean min–max normalized hippocampus-to-cortex ratio	0.37	0.33	0.19	0.26	0.18	—	—
Mean min–max normalized total grey matter volume	0.58	0.79	0.62	0.98	0.36	—	—
Total WMH volume (ICV adjusted)	7.7 (9.4)	8.7 (11)	7 (4.6)	3.1 (0.5)	8.9 (10.3)	3.67 (4)	0.630

The values shown in the table are the means with standard deviations in brackets except for number of individuals, women and *APOE* ɛ4 for which the percentages are provided. χ^2^ tests were used for categorical variables and Kruskal–Wallis for continuous variables and were corrected for multiple comparison with the Holm–Šidák method. Footnotes indicate cases where *P*-values were significant in the *post hoc* pairwise comparisons across AD subtypes, *P* < 0.05. The CN group data are displayed for reference.

*APOE* ɛ4, apolipoprotein E ɛ4 allele; MMSE, Mini-Mental State Examination; CDR, The Clinical Dementia Rating Scale; ADNI-EF, Alzheimer’s Disease Neuroimaging Initiative executive function composite score; ADNI-MEM, Alzheimer’s Disease Neuroimaging Initiative memory composite score; ADNI-LAN, Alzheimer’s Disease Neuroimaging Initiative language composite score; CSF t-tau, total tau; CSF p-tau, phosphorylated tau; CSF Aβ, amyloid-beta 1–42 peptide; WMH, white matter hyperintensity; ICV, estimated total intracranial volume.

^a^Diffuse < Minimal, *P* < 0.05.

^b^Cortical Predominant < Minimal, *P* < 0.05.

^c^Diffuse < Limbic Predominant, *P* < 0.05.

^d^Diffuse+ < Minimal, *P* < 0.05.

^e^Minimal < Cortical Predominant, *P* < 0.05.

^f^Limbic Predominant < Cortical Predominant, *P* < 0.05.

^g^Diffuse < Cortical Predominant, *P* < 0.05.

^h^Minimal < Diffuse, *P* < 0.05.

^i^Limbic Predominant < Diffuse, *P* < 0.05.

^j^Limbic Predominant < Minimal, *P* < 0.05.

^k^Diffuse+ < Cortical Predominant, *P* < 0.05.

^l^Diffuse+ < Limbic Predominant, *P* < 0.05.

### Individual level subtype allocations

**Figure 2:**
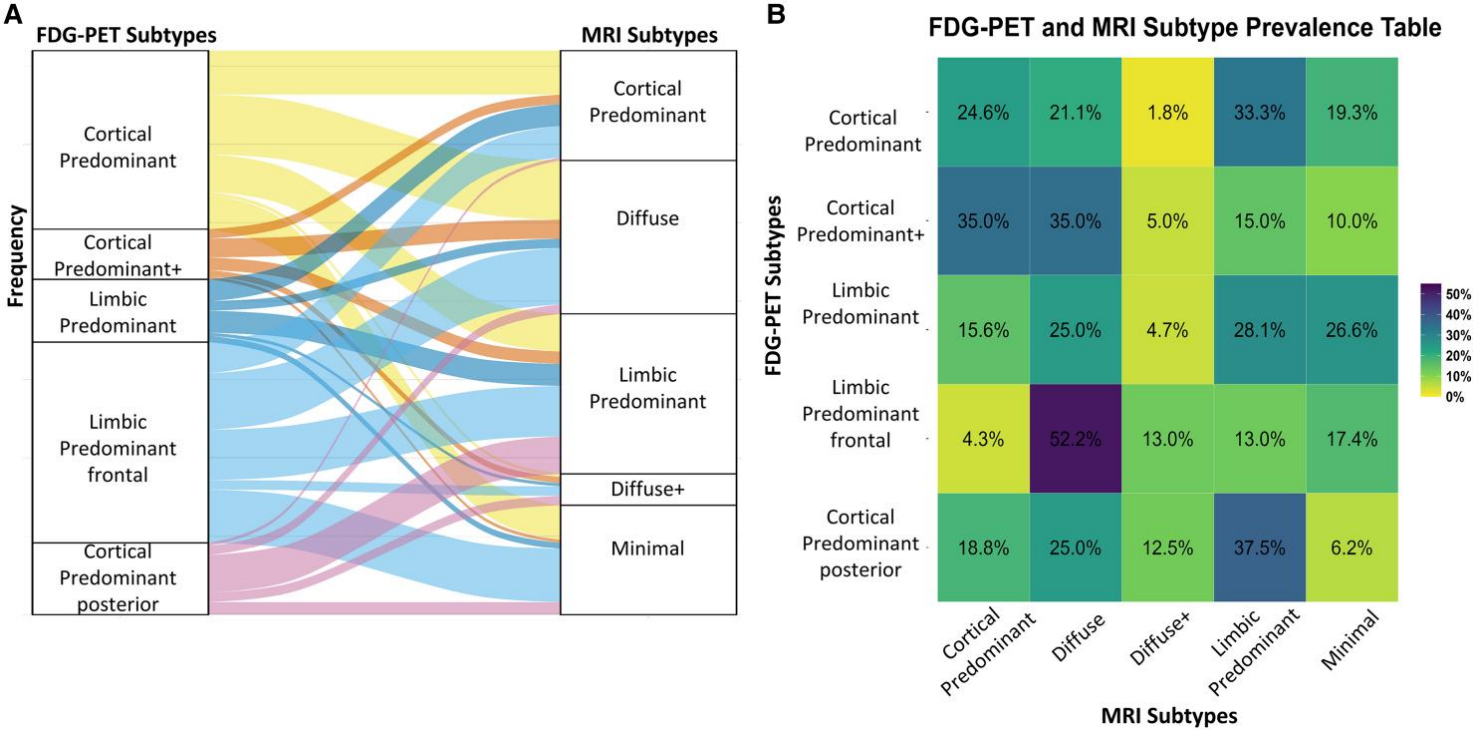
**Relationship between AD subtypes in FDG-PET and MRI at an individual level.** (**A**) Alluvial plot showing the individual level allocation of the FDG-PET and MRI subtypes (frequency on *y*-axis). (**B**) Percentage combination of FDG-PET (*y*-axis) and MRI (*x*-axis) subtypes calculated using total number of each FDG-PET subtype (total percentage sum up row-wise).

To assess the consistency between the two modalities in subtype assignments, the subtype categorizations for individuals were compared across both FDG-PET and MRI (Fig. 2). We propose that the cortical subtypes and limbic subtypes are most similar between the FDG-PET and MRI subtypes. Namely, the FDG-PET Cortical Predominant, Cortical Predominant posterior and Cortical Predominant+ subtypes are equivalent to MRI Cortical Predominant, Diffuse or Diffuse+ patterns topographically, whereas FDG-PET Limbic Predominant and Limbic Predominant frontal are equivalent to MRI Limbic Predominant. As a Minimal pattern was only found in MRI, we do not think that this subtype has an equivalent in FDG-PET.

The agreement between the FDG-PET and MRI subtype allocations was low as this was less than 50%. Although the compared subtypes showed similar topographies of neurodegeneration (i.e. cortical-/limbic-predominant hypometabolism and atrophy, respectively), they did not match at the individual level. All possible combinations of allocated FDG-PET and MRI subtypes of varying percentages were found (Fig. 2A and B). AD individuals classified into the FDG-PET Cortical Predominant subtype matched best with the MRI Limbic Predominant (33.3%) and MRI Cortical Predominant (24.6%) subtypes. In contrast, individuals classified as FDG-PET Cortical Predominant+ matched best with MRI Cortical Predominant (35%) and Diffuse (35%). FDG-PET Limbic Predominant best matched with the three MRI subtypes: Limbic Predominant (28.1%), Minimal (26.6%) and Diffuse (25%). FDG-PET Limbic Predominant frontal best matched with MRI Diffuse (52.2%). FDG-PET Cortical Predominant posterior matched best with MRI Limbic Predominant (37.5%).

### Data-driven versus hypothesis-driven subtyping

**Figure 3:**
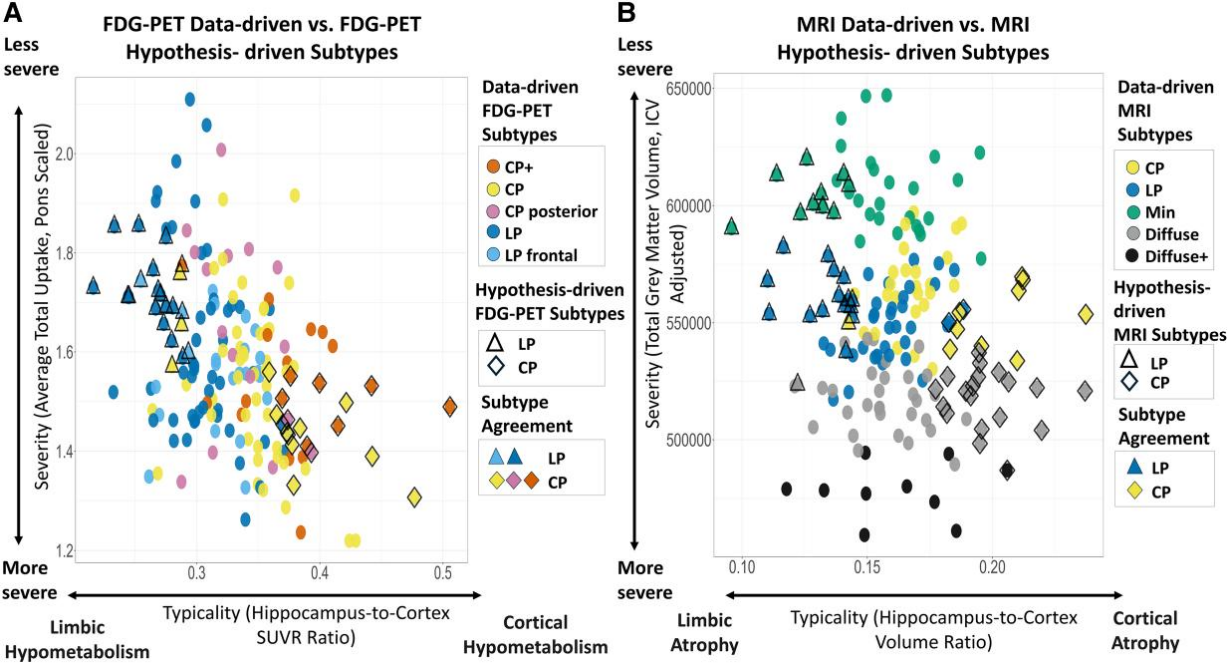
**Data-driven versus hypothesis-driven AD subtypes in FDG-PET and MRI along typicality and severity axes.** Hypothesis-driven subtypes are defined along the ‘typicality’ axis using prior hippocampus-to-cortex ratio classification using grey matter volumes and FDG-PET SUVRs. Each datapoint represents an individual included in this study that was allocated into the data-driven and/or hypothesis-driven subtypes along axes of ‘typicality’ and ‘severity’. For agreement across these two subtyping measures refer to the legend, ‘Subtype Agreement’. (**A**) Data-driven FDG-PET subtypes with FDG-PET hypothesis-driven subtypes along MRI typicality and severity. (**B**) Data-driven MRI subtypes with MRI hypothesis-driven subtypes along MRI typicality and severity. CP, Cortical Predominant; CP+, Cortical Predominant+; LP, Limbic Predominant; LP frontal, Limbic Predominant frontal; Min, Minimal atrophy.

Clustering-based and prior hypothesis-based subtypes were compared within the framework of typicality and severity^2^ in both FDG-PET and MRI (Fig. 3). Within each modality, data-driven and hypothesis-driven subtypes overlapped with each other reasonably well for most subtypes (Fig. 3A and B). The agreement between MRI data-driven and MRI hypothesis-driven limbic-predominant subtypes (55.6%) was better than that between cortical-predominant subtypes (30%) (Fig. 3B). Contrarily, the agreement between FDG-PET data-driven and FDG-PET hypothesis-driven cortical-predominant subtypes (90%) was better than that between limbic-predominant subtypes (82%) (Fig. 3A).

**Figure 4:**
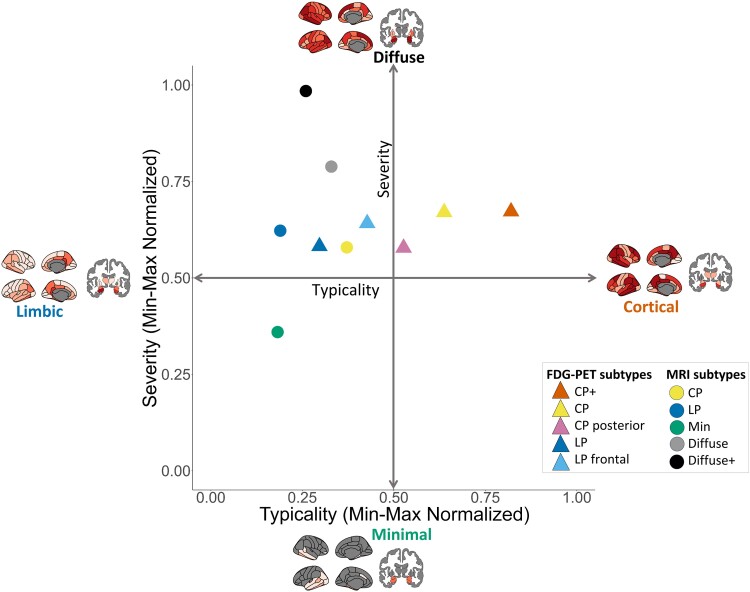
**Data-driven neurodegeneration-based AD subtypes along axes of typicality and severity**. The current study’s neurodegeneration-based AD subtypes were defined along the typicality–severity framework (Ferreira *et al*., 2020). The placement of the subtypes was calculated using the mean min–max normalized ‘typicality’ and ‘severity’ values for the subtypes (SUVR-based values for FDG-PET subtypes, volume-based for MRI subtypes). Each datapoint represents the mean min–max normalized value per subtype; values are also given in Tables 2 and 3. The colours correspond to a neurodegeneration AD subtype identified in the current study. CP, Cortical Predominant; CP+, Cortical Predominant+; LP, Limbic Predominant; LP frontal, Limbic Predominant frontal; Min, Minimal atrophy.

Association between typicality and severity by modality differed. Correlation between typicality and severity (Table 4) was significant in FDG-PET (*R*^2^ = 0.25, *P* < 0.001), but not in MRI. The severity measures (*R*^2^ = 0.15, *P* < 0.001) in FDG-PET and MRI were more strongly associated with each other than typicality measures (*R*^2^ = 0.02, *P* < 0.05). Additionally, FDG-PET subtypes are more separable across the typicality axis than MRI, which is evident when comparing averaged normalized values of typicality and severity for each subtype (Fig. 4), whereas for MRI subtypes, there was a clearer split of the along the severity axis.

**Table 4:** Correlations between typicality and severity in FDG-PET and MRI

Model	R²	*P*-values
FDG-PET typicality and FDG-PET severity (Fig. 3A)	0.25	< 0.001**
MRI typicality and MRI severity (Fig. 3B)	0.0011	0.53
FDG-PET severity and MRI severity	0.15	<0.001**
FDG-PET typicality and MRI typicality	0.02	0.0073*

Pearson’s correlations between measures of ‘typicality’ and ‘severity’ in both modalities.

^*^ *P* < 0.05.

^**^ *P* < 0.001.

## Discussion

This study investigated cross-modality AD subtypes by applying data-driven subtyping models to regional FDG-PET and MRI data. To our knowledge, this is the first study to implement identical data-driven models to concurrent FDG-PET and MRI data to identify and compare modality-specific neurodegeneration-based AD subtypes. Despite FDG-PET and MRI both being interchangeable measures of neurodegeneration within the ATN framework,^45,46^ our findings show that the respective neurodegeneration subtypes differed across modalities and within individuals.

Using the same data-driven model, cortical and limbic AD subtypes were independently identified in both FDG-PET and MRI methodology. At a group level, the expected pattern of temporo-parietal hypometabolism^50-54^ was found in the AD group. However, FDG-PET subtypes showed distinct patterns of hypometabolism: Cortical Predominant, Cortical Predominant+, Cortical Predominant posterior, Limbic Predominant and Limbic Predominant frontal. Our findings resemble findings of the only previous study addressing FDG subtypes in AD.^11^ In terms of topography, Cortical Predominant, Cortical Predominant posterior and Limbic Predominant subtypes in our study are closest to the Typical subtype (hypometabolism in cortical and limbic regions) identified by Levin *et al*.^11^ although with differing clinical characteristics. The differences in findings could possibly be explained by methodological (clustering method, regions used for clustering, etc.) and sample differences. Two subtypes that were identified included the Cortical Predominant+ (younger, fewer *APOE* ɛ4 carriers, executive function impairment) and Limbic Predominant frontal (older age, more *APOE* ɛ4 carriers), which align well with the Cortical Predominant and Limbic Predominant subtypes, respectively, identified by Levin *et al*.^11^ Together, these two studies indicate the clear presence of distinct cortical- and limbic-predominant profiles of hypometabolism in AD. In addition, our supplementary findings using the MCI sample also showed a presence of cortical versus limbic subtypes at a prodromal stage with more diluted patterns of neurodegeneration.

The MRI pattern of the whole AD group showed the expected pattern of medial temporal and hippocampal atrophy.^55-58^ However, the MRI AD subtypes showed five distinct patterns: Cortical Predominant, Limbic Predominant, Minimal atrophy, Diffuse and Diffuse+. Our data-driven MRI subtypes had similar percentages and clinical presentation to what has been found previously.^2,9,43,59,60^ In accordance with previous studies, Limbic Predominant had focal limbic atrophy and later age at onset. Cortical Predominant resembles the ‘Hippocampal Sparing’ MRI subtype previously described, with greater atrophy in the cortical relative to the limbic regions, as well as greater executive function impairment, higher proportion of men and younger age. The two Diffuse subtypes in this study resemble Typical and Diffuse subtypes identified previously (widespread atrophy, older and worse memory scores). At the other end of the severity dimension, Minimal atrophy subtype resembled prior Minimal subtypes topographically and clinically (shortest disease duration and highest MMSE). Our MCI findings also identified minimal and limbic subtypes with diminished patterns of neurodegeneration, but a cortical pattern was not identified this early in the disease stage.

We did not observe an overlap in terms of topography nor demographics, despite identifying cortical and limbic subtypes in both FDG-PET and MRI. As the ‘typical’ AD pattern for each modality differs, this can be seen in the subtypes’ modality-specific neurodegeneration. Key regions show neurodegeneration at varying levels of severity, such as posterior cingulate cortex in FDG-PET (Fig. 1C) and medial temporal lobe and hippocampus for MRI (Fig. 1D). FDG-PET subtypes show a clearer cortical pathway compared with the MRI subtypes, which are more susceptible to more limbic neurodegeneration. These results are in line with the common AD patterns in these two modalities: neocortical hypometabolism in FDG-PET^51,53,61^ and medial temporal atrophy in MRI.^55,57^ While the regional atrophy does not mimic hypometabolism in FDG-PET subtypes (Fig. 1C and E), regional hypometabolism mirrors atrophy in MRI subtypes more closely (Fig. 1D and F). For example, cortical-predominant FDG-PET subtypes with limited hippocampal hypometabolism were found to show considerable limbic (hippocampal) atrophy. This finding is congruent with the well-established evidence that higher cortical hypometabolism is closely associated with higher hippocampal atrophy.^17,19,62^ Our study goes a step further to demonstrate that in contrast, a cortical-predominant MRI subtype with limited hippocampal atrophy also shows cortical-predominant hypometabolism with limited hippocampal hypometabolism. A previous study assessed FDG-PET severity within AD typical cortical regions in hypothesis-driven MRI subtypes using the hypometabolic convergence index.^7^ Hippocampal Sparing had highest values of this index compared with Limbic Predominant and Typical AD subtypes, highlighting the overlap in cortical hypometabolism and atrophy. Although our FDG-PET subtypes had similar patterns of cortical hypometabolism and atrophy, this was not the case when assessing deeper structures (Fig. 1E and F). Thus, FDG-PET and MRI do not necessarily share a bidirectional relationship in capturing hippocampal neurodegeneration. A previous study comparing tau-PET and MRI subtypes longitudinally also showed that the modalities do not always correspond, and atrophy is not always a downstream event. This was particularly true for the more atypical subtypes.^24^ Further, it would be interesting to validate a similar method across tau-PET and FDG-PET subtypes in future work.

Interestingly, when assessing how individuals were subtyped in both modalities, a remarkably low classification correspondence between equivalent subtypes (i.e. cortical-/limbic- predominant) in the different modalities was found (Fig. 2A). Individuals classified as cortical-predominant hypometabolism subtypes were not always classified as cortical-predominant atrophy subtypes and even matched with limbic-predominant atrophy patterns. Limbic hypometabolism subtypes agreed with different levels of atrophy severity from Minimal to Diffuse. The ‘disconnection hypothesis’ has been proposed in AD to explain concurrent retrosplenial cortex hypometabolism and medial temporal atrophy.^15,19,63^ It has been proposed that local and/or distant atrophy results in downstream hypometabolism in the parietal and retrosplenial cortex.^19,64^ In our corresponding neurodegeneration maps (Fig. 1E and F), association between levels of isthmus cingulate (part of retrosplenial cortex) hypometabolism relative to medial temporal lobe atrophy can be appreciated. FDG-PET subtypes; Cortical Predominant and Cortical Predominant posterior had greatest isthmus cingulate hypometabolism and consequently displayed greater medial temporal atrophy than the other subtypes (Fig. 1C and E). Conversely, Cortical Predominant+ hypometabolism subtype had greatest atrophy in cingulate/precuneus regions and least in the hippocampus compared with the other hypometabolism subtypes. This is in concordance with prior findings in early-onset AD patients who typically show more posterior cortical atrophy compared with hippocampal atrophy.^61,65^ MRI subtypes’ corresponding hypometabolism maps all showed isthmus cingulate involvement even in the Minimal atrophy subtype (Fig. 1D and F). This finding resembles prior findings of minimal hippocampal and posterior cingulate atrophy AD pattern that showed significant hypometabolism.^61^ This could be indicative of FDG-PET being an earlier marker of neurodegeneration than MRI.^66-69^ Due to the poor compatibility between FDG-PET and MRI AD subtypes, a combined model may be more suitable for subtyping and should be tested in future studies. Moreover, neuropathological data and their association with specific neurodegeneration patterns could provide the missing link between FDG-PET and MRI. Potential copathologies are often present in combination with AD;^70^ therefore, more analysis is needed especially within subtypes^60^ and across different imaging modalities.^71^ Different pathologies have been linked to specific patterns of atrophy and hypometabolism.^72-74^ These copathologies could also explain the divergence between atrophy and metabolism patterns.

Measures such as the hippocampus-to-cortex ratio based on neuropathological data^5^ have been applied successfully using grey matter volumes from MRI.^6,7^ In this study, we compared such measures with findings from our data-driven approach.^2^ Our findings showed that there was agreement between hypothesis-driven and data-driven approaches. Interestingly, FDG-PET Cortical Predominant+ also had higher hippocampus-to-cortex ratio when using grey matter volumes similar to the finding in Levin *et al*.^11^. The data-driven subtypes additionally capture the second axis of ‘severity’, as Minimal and Diffuse subtypes were found in the MRI. It can also be argued that our FDG-PET subtypes show different levels of ‘severity’ as Limbic Predominant frontal and Cortical Predominant+ show greater overall hypometabolism than the other limbic and cortical subtypes. In a clinical setting, a simplified version of the subtyping may be preferred for example using visual ratings of atrophy and/or hypothesis-driven subtyping (typicality and severity) as shown in this study. However, the contrast between data-driven and hypothesis-based methods highlights the issue with harmonization of methods for subtyping in AD.^4^ In general, hypothesis-driven hippocampus-to-cortex ratios work well using FDG-PET SUVRs and match well with data-driven subtypes. However, as this is the first study to test these two methods using FDG-PET SUVRs, this would need to be further validated. Typicality and severity were correlated in FDG-PET indicating that cortical subtypes are the more severe compared with the limbic subtypes, whereas typicality and severity were not correlated in MRI, showing that the link is different across the two modalities (Table 4). These findings could indicate that there are more complex mechanisms at play, such as copathologies^72,74^ as neither of these measures are specific to AD.

Conceptually, FDG-PET and MRI subtypes found in our study can be defined along the ‘typicality’ and ‘severity’ axes (Fig. 4). Typicality splits the two modality-specific subtypes into either a limbic or cortical pattern. Severity splits the extremes found in the MRI subtypes of limited to widespread atrophy. In contrast, there was a greater split along the typicality axis for FDG-PET subtypes between cortical and limbic hypometabolism. In this study, we propose that subtypes do not always lie orthogonal along these two axes and are often a combination of both. Namely, we found that FDG-PET Limbic Predominant subtype is left of the centre towards limbic along the typicality axis but is positioned lower on the severity axis compared with Limbic Predominant frontal. The same applies to the Cortical Predominant+ and Cortical Predominant subtypes which have widespread hypometabolism. The severity for the current FDG-PET subtypes is backed by frontal hypometabolism being indicative of a later stage of AD.^75^ The complexity of the relationship of the two modalities is reflected in this figure, and underlying mismatches between the two modalities have been highlighted throughout this study.

This study has various limitations; one is its cross-sectional design. To concretize the current findings, longitudinal studies are needed to be able to label these patterns as subtypes. Longitudinal clustering has been performed in AD to investigate heterogeneity of topographical differences in MRI.^9,76^ In addition, tau- and amyloid-PET studies have used cross-sectional data with a probabilistic model for predicting disease subtype and stage.^77,78^ Future research should explore the progression of glucose metabolism across these subtypes in relation to other imaging modalities. Additionally, testing the models and classification of the subtypes in clinical cohorts for external validity is important. The samples used in the current study are mainly amnestic individuals due to ADNI’s strict inclusion/exclusion criteria. This limits the assessment of the overlap between the biological subtypes observed in this study and atypical AD syndromes. Another limitation is that the individuals in this study likely have mixed pathologies that cannot be detected through neuroimaging alone. Utilizing neuropathological data in combination with *in vivo* data should be a focus in future studies.

Ultimately, the current study identified data-driven cortical and limbic AD subtypes from FDG-PET and MRI scans. These data-driven subtypes overlapped well with hypothesis-driven methods, validating our findings. Although, the main finding was that structure does not always reflect function when assessing corresponding patterns of neurodegeneration in these subtypes. Cortical and limbic subtypes did not overlap in the two modalities in terms of individual subtype allocation. Furthermore, these subtypes lie along ‘severity’ and ‘typicality’ axes distinctly across modalities as shown in our conceptual figure (Fig. 4). Copathologies may contribute to this divergence in FDG-PET and MRI subtype patterns. In conclusion, the current findings highlight the need for a multimodal perspective for understanding the complex biological AD mechanisms.

## Supplementary Material

fcae426_Supplementary_Data

## Data Availability

The data used in this study are available from the corresponding authors on request. Raw data are available from the LONI database (https://ida.loni.usc.edu).
