# Supplementary material for: Divergent neurodegenerative patterns: Comparison of [18F] fluorodeoxyglucose-PET- and MRI-based Alzheimer’s disease subtypes
Source: Brain Commun. 2024 Nov 23;6(6):fcae426. doi: 10.1093/braincomms/fcae426 (PMC11656166; doi:10.1093/braincomms/fcae426)
Supplement: fcae426_Supplementary_Data [file fcae426_supplementary_data.zip › Supplementary_material.docx]

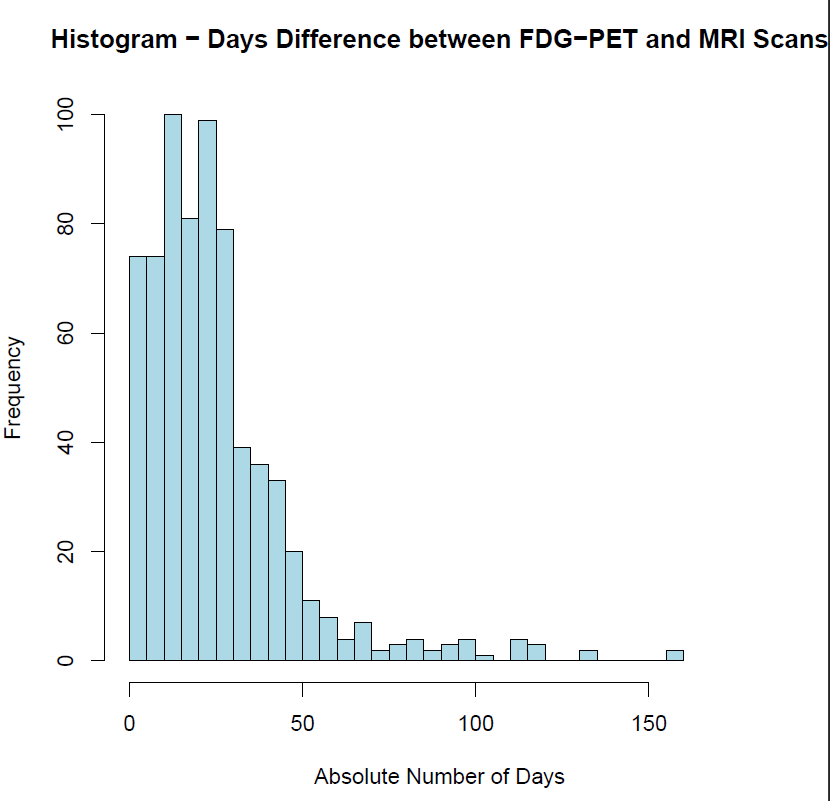


**Supplementary Figure 1: Histogram of absolute number of days between FDG-PET and MRI scans.** The counts of the absolute number of days between initial scans used in this study were plotted. The mean number of absolute days was 25 days. In the current sample, initial FDG-PET scans were performed: prior (10%), after (85%), and same day (5%) as MRI scans.

|  |  | **FDG Subtypes** | | | | | **MRI Subtypes** | | | | |
| --- | --- | --- | --- | --- | --- | --- | --- | --- | --- | --- | --- |
|  | Cognitively Normal (N=176) | Cortical Predominant (N=57) | Cortical Predominant+ (N=20) | Limbic Predominant (N=64) | Limbic Predominant frontal (N=23) | Cortical Predominant posterior (N=16) | Cortical Predominant (N=35) | Diffuse (N=51) | Limbic Predominant (N=49) | Diffuse+ (N=10) | Minimal (N=35) |
| Age | 0 | 0 | 0 | 0 | 0 | 0 | 0 | 0 | 0 | 0 | 0 |
| Sex | 0 | 0 | 0 | 0 | 0 | 0 | 0 | 0 | 0 | 0 | 0 |
| Education | 0 | 0 | 0 | 0 | 0 | 0 | 0 | 0 | 0 | 0 | 0 |
| Disease Duration | — | 1 | 0 | 4 | 0 | 2 | 2 | 0 | 1 | 1 | 3 |
| Age at Onset | — | 1 | 0 | 4 | 0 | 2 | 2 | 0 | 1 | 1 | 3 |
| MMSE | 20 | 6 | 3 | 6 | 1 | 2 | 4 | 4 | 8 | 1 | 1 |
| Global CDR | 19 | 6 | 3 | 6 | 1 | 2 | 4 | 4 | 8 | 1 | 1 |
| ADNI-EF | 0 | 0 | 0 | 1 | 0 | 0 | 0 | 1 | 0 | 0 | 0 |
| ADNI-MEM | 0 | 0 | 0 | 1 | 0 | 0 | 0 | 1 | 0 | 0 | 0 |
| ADNI-LAN | 0 | 0 | 0 | 1 | 0 | 0 | 0 | 1 | 0 | 0 | 0 |
| APOE ɛ4 | 0 | 0 | 0 | 0 | 0 | 0 | 0 | 0 | 0 | 0 | 0 |
| CSF t-tau | 67 | 12 | 5 | 16 | 2 | 3 | 5 | 7 | 14 | 4 | 8 |
| CSF p-tau | 67 | 12 | 5 | 16 | 2 | 3 | 5 | 7 | 14 | 4 | 8 |
| CSF Aβ | 67 | 12 | 5 | 16 | 2 | 3 | 5 | 7 | 14 | 4 | 8 |
| White Matter Hyperintensity Volume | 43 | 11 | 3 | 19 | 10 | 2 | 6 | 23 | 10 | 6 | 0 |

**Supplementary Table 1:** Missing baseline collected values for demographic, clinical and biomarker data in cognitively normal and AD subtypes.

**
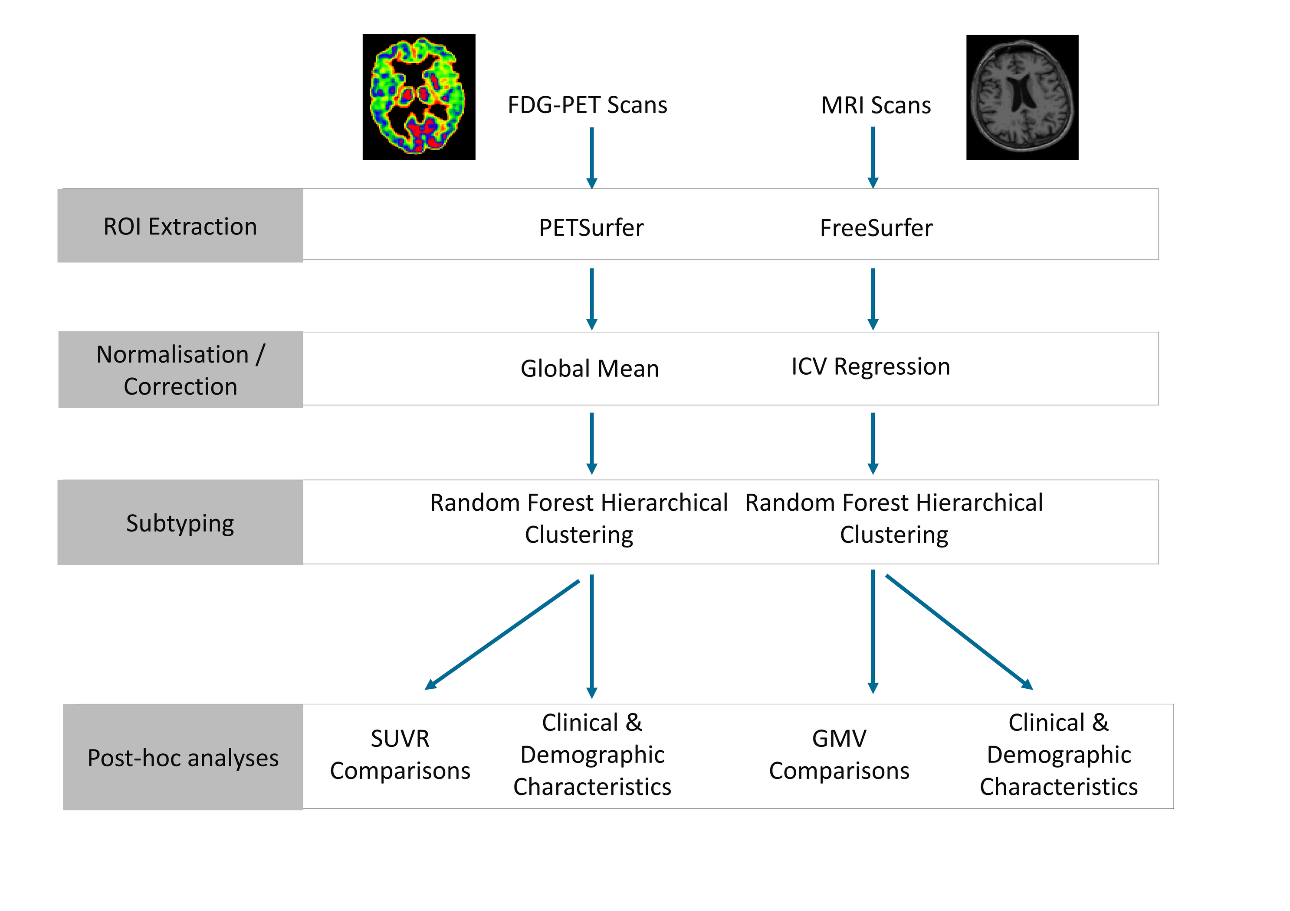
Supplementary Figure 2: Experimental Pipeline.** Steps carried out for each modality in parallel. Regional values were extracted using PETSurfer and FreeSurfer version 6.0.0. Intensity normalisation using the global mean per individual for FDG-PET values and for MRI, head-size correction was carried out. These data were then used for the random forest hierarchical clustering (Supplementary Figure 3 for the full pipeline). Once groups were defined, they were characterised by biological, clinical and demographic information. *Abbreviations*: ICV: intracranial volume, SUVR: standard uptake value ratio, GMV: grey matter volume.


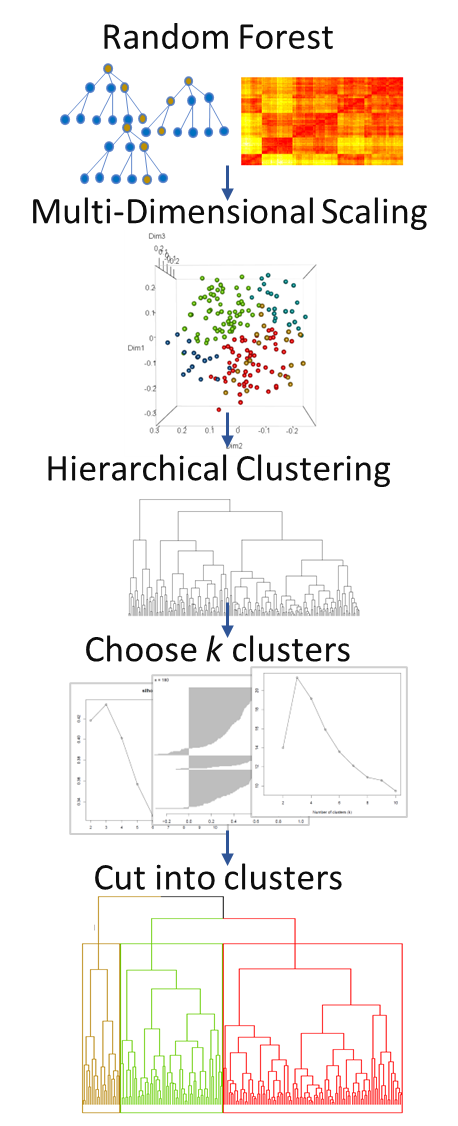


**Supplementary Figure 3:** **Random forest hierarchical clustering pipeline.** The steps carried out for the random forest clustering method on regional FDG-PET SUVR and MRI grey matter volumes. Random forest models were run using regional data which was then reduced to lower dimensionality. This output was then used for the agglomerative hierarchical clustering. The number of clusters was selected using various cluster validation indices and groups were cut with the selected number.

**
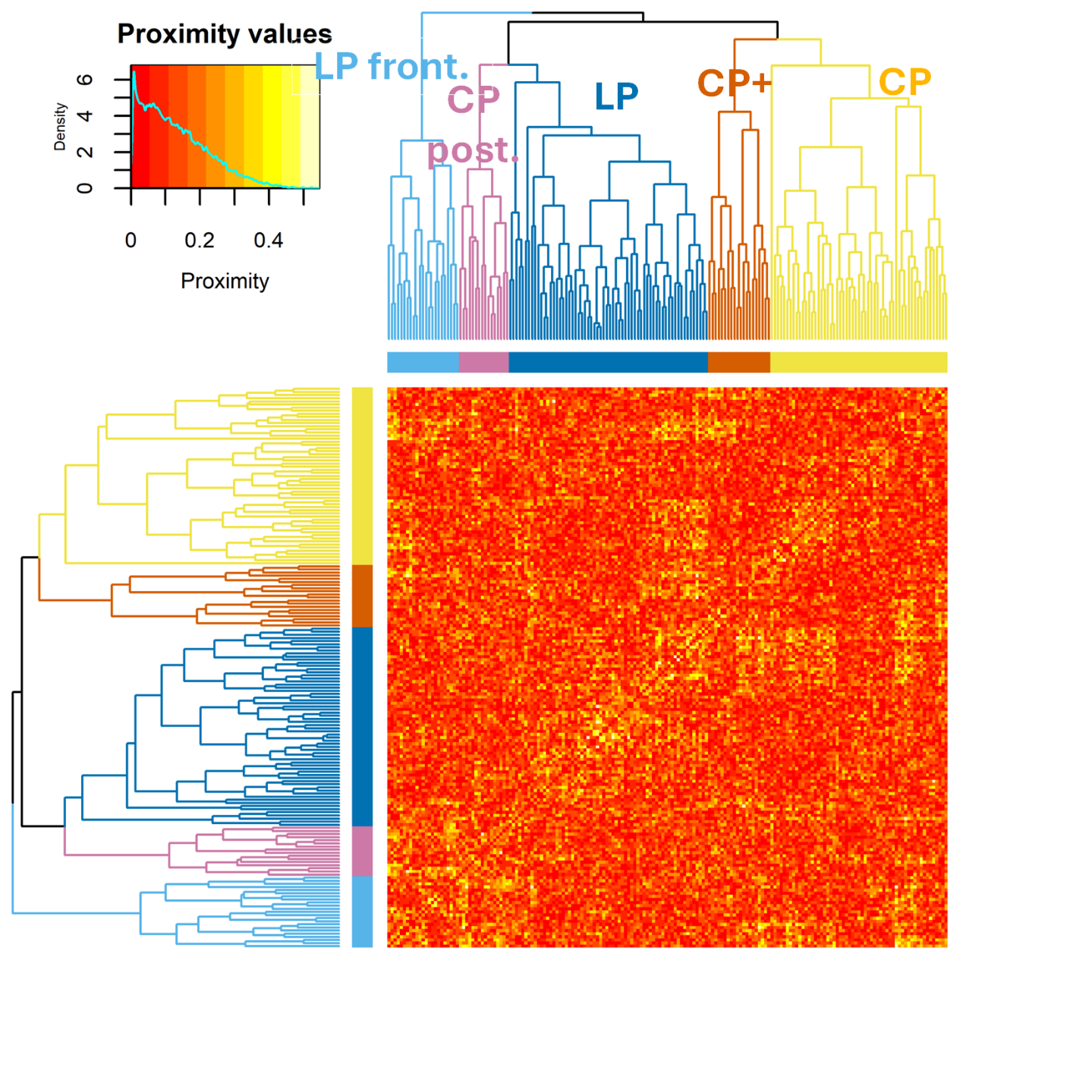
**

**Supplementary Figure 4:** **Proximity matrix assessment of the FDG-PET clustering model.** The histogram in the upper left corner represents a scale from 0 to 1 for the average difference between the proximity matrix and 100 simulated random forest matrices. The heatmap shows the similarity within and between clusters with the corresponding dendrogram. Within the heatmap are the proximity differences whereby lower values indicate more stability in red and higher values indicate less stability in yellow. Overall, the heatmap shows that the model is robust and stable.

**
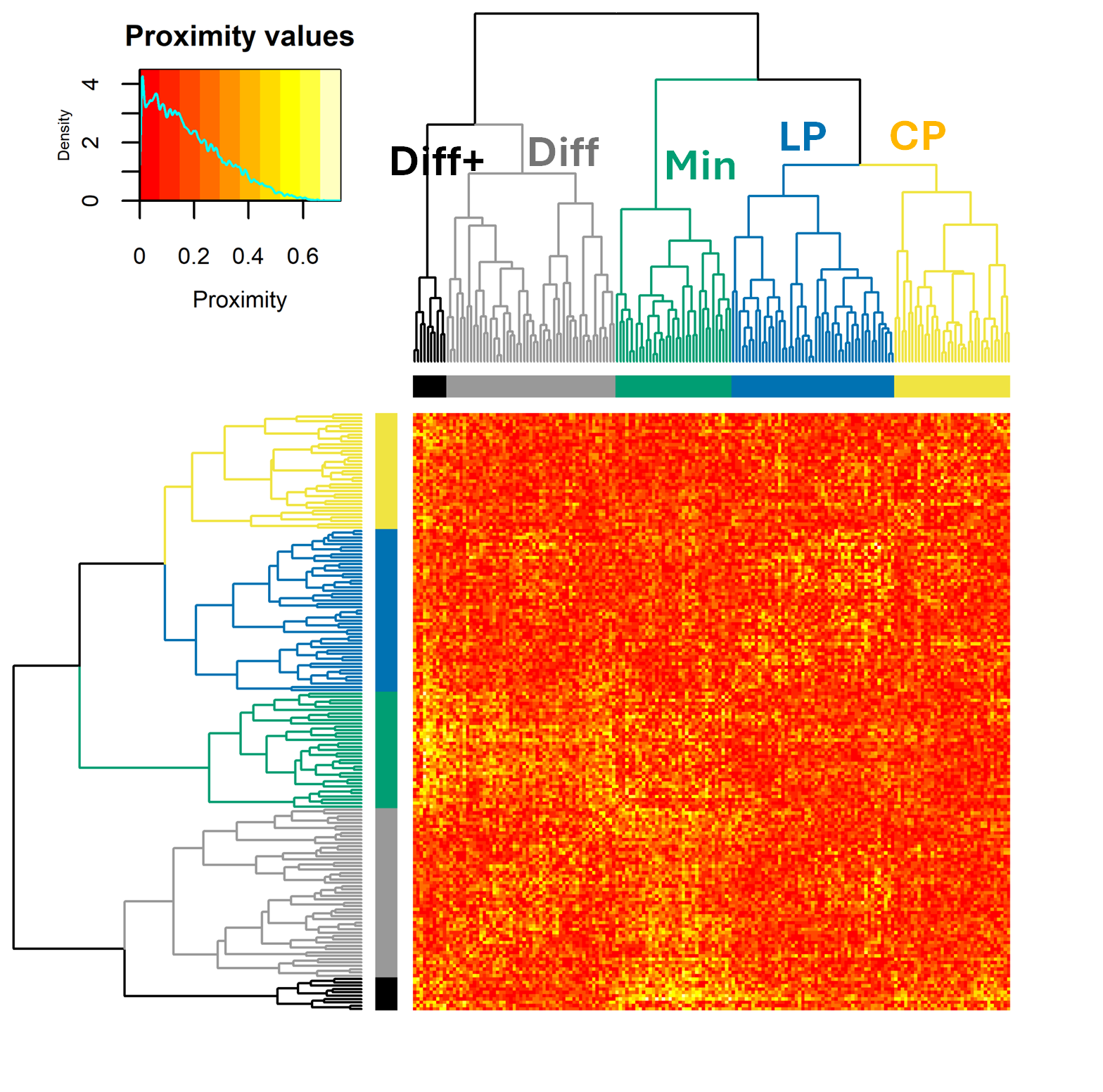
 Supplementary Figure 5:** **Proximity matrix assessment of the MRI clustering model.** The histogram in the upper left corner represents a scale from 0 to 1 for the average difference between the proximity matrix and 100 simulated random forest matrices. The heatmap shows the similarity within and between clusters with the corresponding dendrogram. Within the heatmap are the proximity differences whereby lower values indicate more stability in red and higher values indicate less stability in yellow. Overall, the heatmap shows that the model is robust and stable. Patches of lesser stability can be observed, especially between Minimal and Diffuse+.


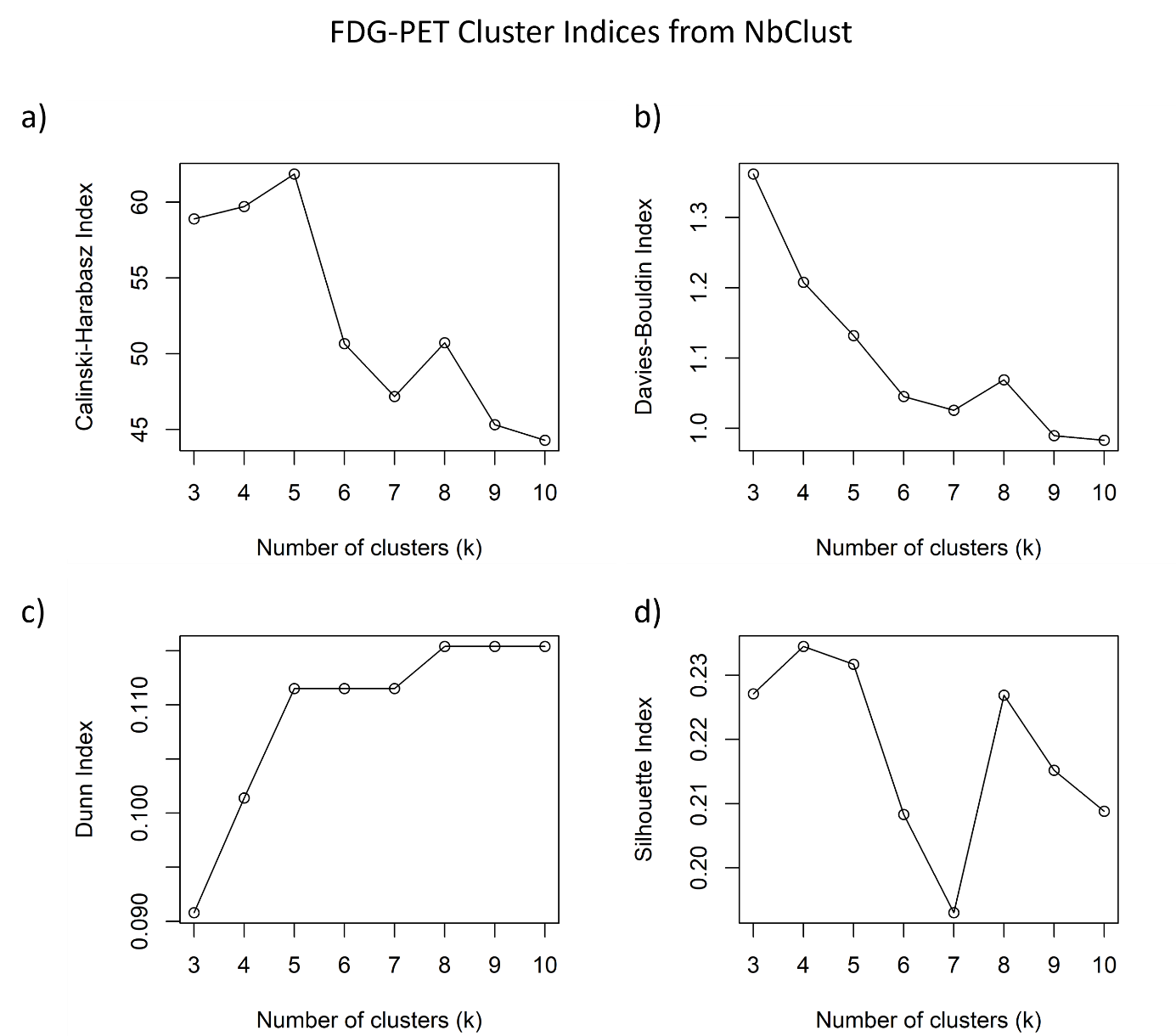


**Supplementary Figure 6: Cluster validation indices’ plots for FDG-PET random forest output.** Four indices were assessed to evaluate the optimal number of clusters for the FDG-PET random forest hierarchical clustering model. Plots display values for: a) Calinski-Harabasz, b) Davies-Bouldin, c) Dunn, d) Silhouette indices from the *NbClust* package in R.


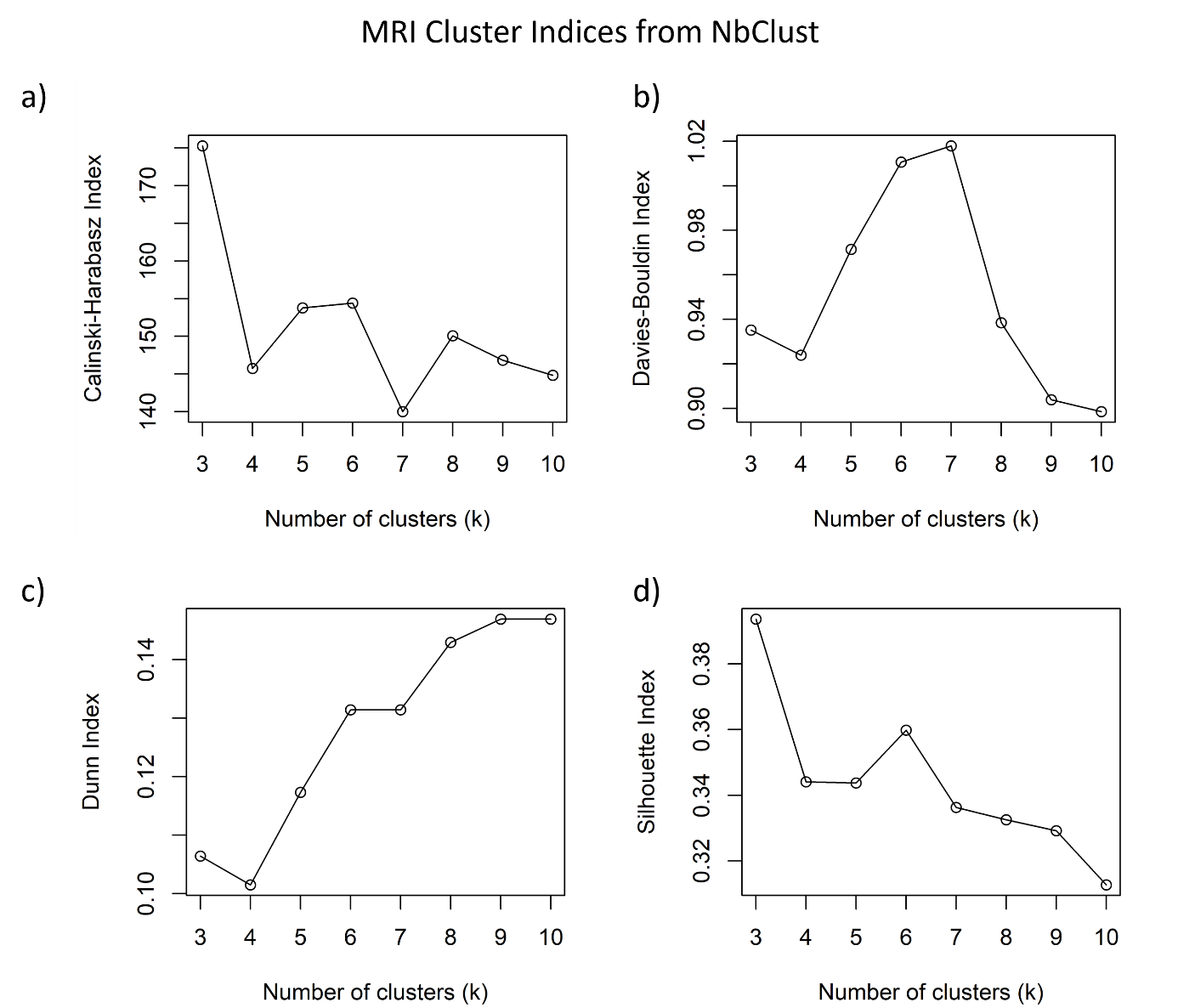


**Supplementary Figure 7: Cluster validation indices plots for MRI random forest output.** Four indices were assessed to evaluate the optimal number of clusters for the MRI random forest hierarchical clustering model. Plots display values for: a) Calinski-Harabasz, b) Davies-Bouldin, c) Dunn, d) Silhouette indices from the *NbClust* package in R.

***
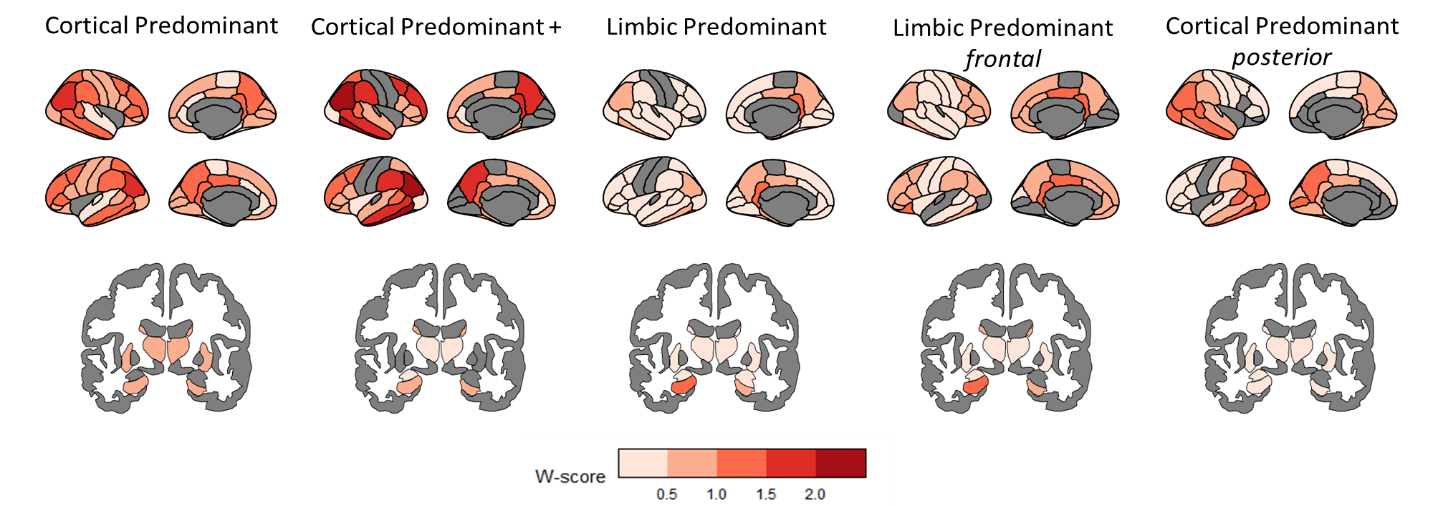
***

**Supplementary Figure 8:** **FDG-PET subtypes’ hypometabolism brain maps**. Brain maps for FDG-PET subtypes using partially volume corrected FDG-PET data with the pons as the reference region for w-score calculation. Brain maps are adjusted for age, sex, education and *APOE* ε4 carriership.


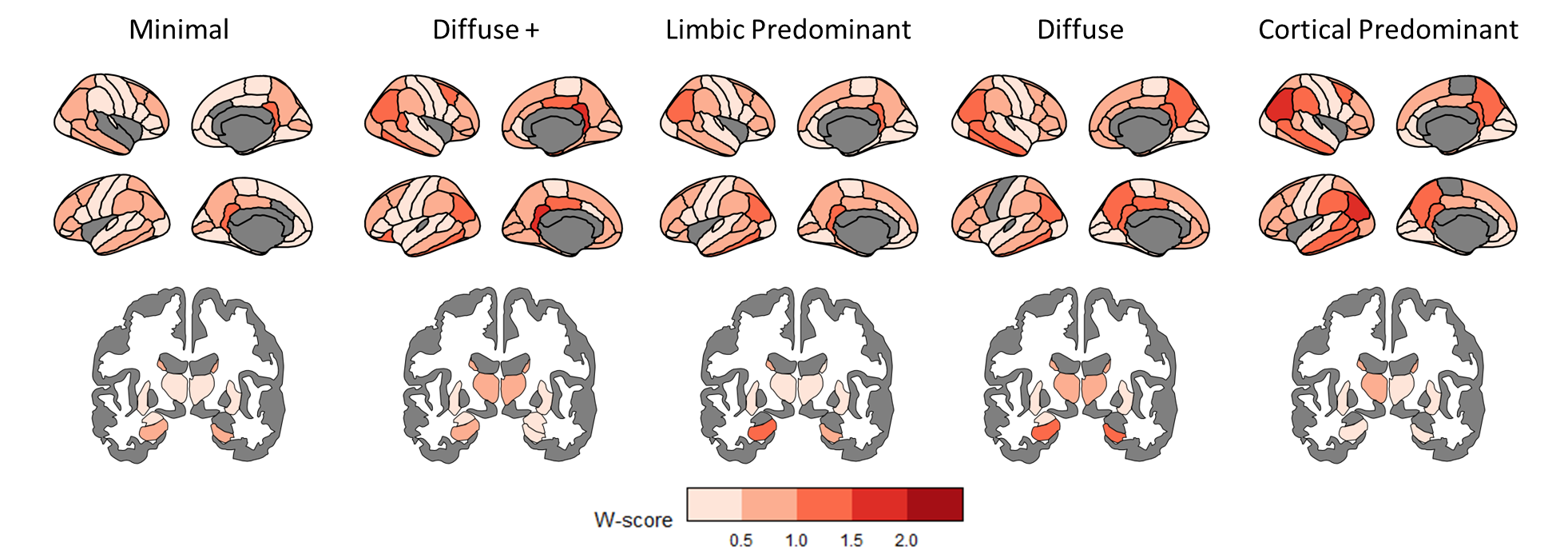


**Supplementary Figure 9:** **MRI subtypes’ corresponding hypometabolism brain maps.** Corresponding hypometabolism brain maps for MRI subtypes using partially volume corrected FDG-PET data with the pons as the reference region for w-score calculation. Brain maps are adjusted for age, sex, education and *APOE* ε4 carriership.


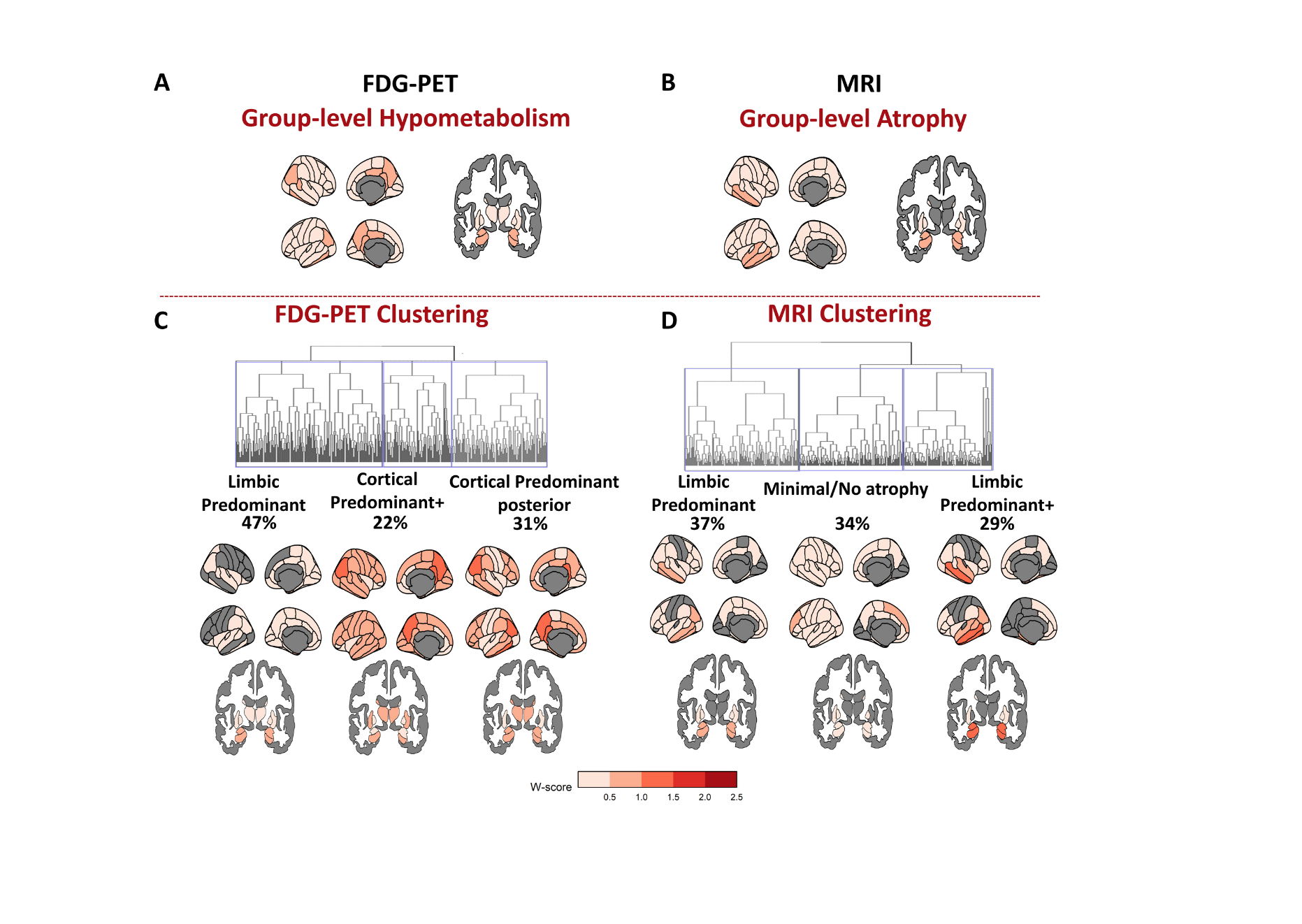


**Supplementary Figure 10:** **Data-driven MCI subtypes based on FDG-PET and MRI.** Overall patterns of neurodegeneration in Aβ+ MCI versus Aβ- CNs visualised for **A)** hypometabolism in FDG-PET and **B)** atrophy in MRI. Clustering identified three subtypes based on: **C)** hypometabolism and **D)** atrophy, which are shown by dendrograms and brain maps in each modality. All brain maps are represented as w-scores where regional values (pons scaled SUVR in FDG-PET and volumes in MRI) are adjusted for age, sex, education and *APOE* ε4 carriership.
